# Efficient synthesis of dihydropyrimidinones via a three-component Biginelli-type reaction of urea, alkylaldehyde and arylaldehyde

**DOI:** 10.3762/bjoc.9.320

**Published:** 2013-12-11

**Authors:** Haijun Qu, Xuejian Li, Fan Mo, Xufeng Lin

**Affiliations:** 1 Department of Chemistry, Zhejiang University, Hangzhou 310027, China

**Keywords:** Biginelli-type reaction, chiral phosphoric acid, dihydropyrimidinone, iodine, multicomponent reaction

## Abstract

A one-pot three-component synthesis of dihydropyrimidinones via a molecular iodine-catalyzed tandem reaction of simple readily available mono-substituted urea, alkylaldehyde, and arylaldehyde has been developed. The reaction proceeds with high chemo- and regioselectivity to give highly diverse dihydropyrimidinones in reasonable yields under mild reaction conditions. Moreover, the first catalytic enantioselective version of this reaction was also realized by using chiral spirocyclic SPINOL-phosphoric acids.

## Introduction

The dihydropyrimidinones (DHPMs) have exhibited interesting and multifaceted biological activities, such as antiviral, antitumor, antibacterial, and antiflammatory properties as well as calcium channel modulating activity [1–2]. As a consequence, the synthesis of dihydropyrimidinone derivatives bearing diverse substitution patterns has attracted significant attention since its discovery 120 years ago in 1893 by the Italian chemist Pietro Biginelli [3–4]. Among them, the Biginelli multicomponent reaction, involving a multicomponent condensation of aldehyde, β-ketoester, and urea, provides an easy access to the preparation of DHPMs, because multicomponent reactions (MCRs) are considered with high facileness, efficiency and economy in organic chemistry [5–8]. Recently, many one-pot variants of Biginelli-type reactions for the preparation of novel DHPMs using various active methylene compounds [9–15], such as enaminone, cyclic β-diketones, acetophenone, benzocyclic ketones and β-oxodithioesters etc., have also been developed to be carried out in the presence of a Lewis or protic acid. It is still highly valuable to develop new direct approaches for the efficient synthesis of DHPMs due to the continued importance of the dihydropyrimidinone core in organic and medicinal chemistry.

Recently, molecular iodine has emerged as an inexpensive, low-toxic catalyst with moderate Lewis acidity and water-tolerance in organic chemistry [16]. Previously, we have developed some molecular iodine-catalyzed organic transformations [17–21], herein we describe the first molecular iodine-catalyzed one-pot three-component Biginelli-type synthesis of DHPMs from simple readily available mono-substituted urea, alkylaldehyde, and arylaldehyde under mild reaction conditions [22–24]. The present method is suitable for a wide range of substrates, and especially for functionalized arylaldehydes. The first catalytic enantioselective version of this reaction is also presented by using chiral spirocyclic SPINOL-phosphoric acids (SPAs) as the catalyst.

## Results and Discussion

**Figure 1:**
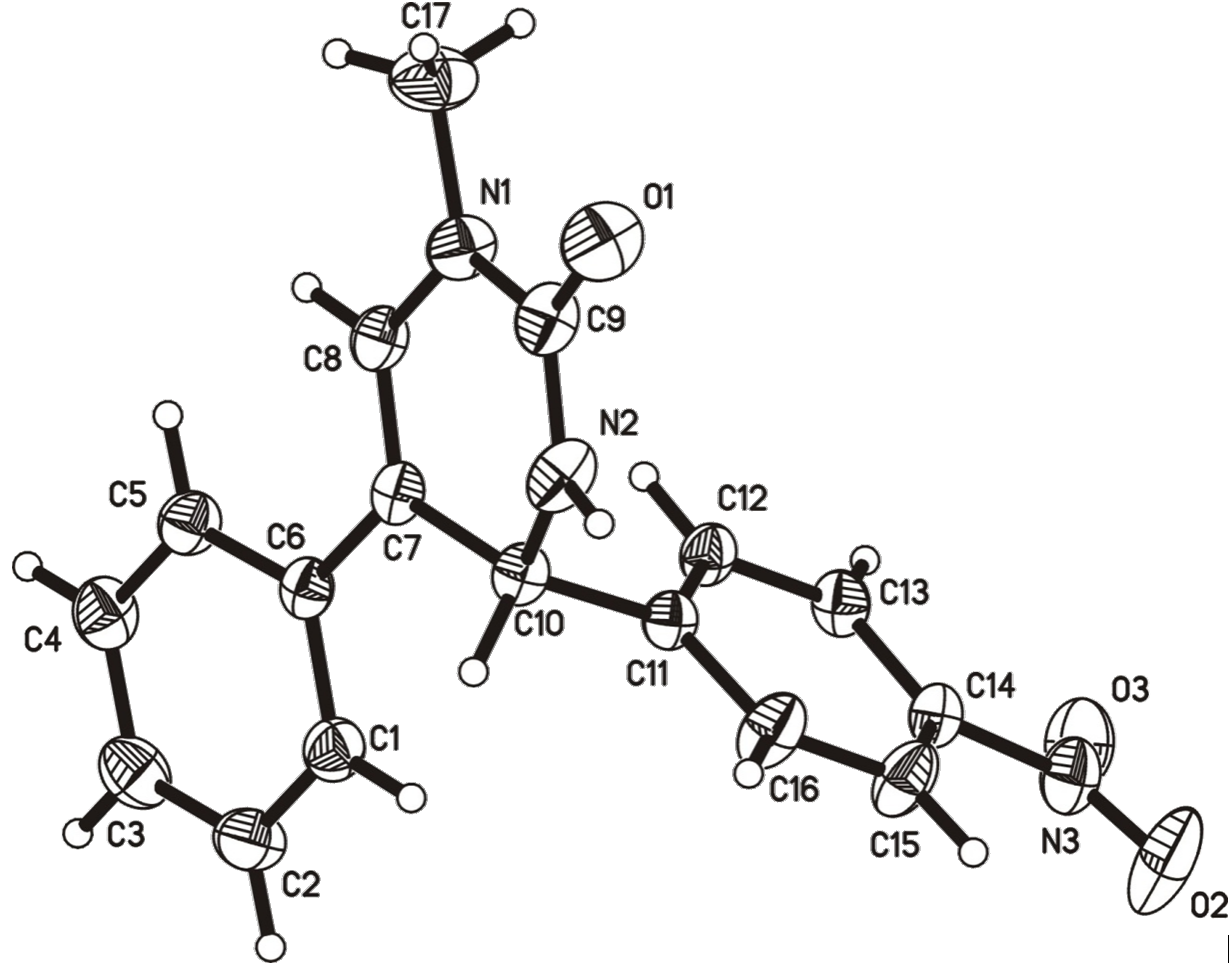
X-ray crystal structure of **4a**.

Initially, the mixture of *N*-methylurea (**1a**, 2.5 mmol), phenylacetaldehyde (**2a**, 2.5 mmol) and *p*-nitrobenzaldehyde (**3a**, 3.75 mmol) in toluene (3 mL) was treated with 10 mol % of iodine under reflux for 12 hours. The functionalized dihydropyrimidinone **4a** was obtained in 56% yield and the structure of the product was clearly assigned by both abundant spectral analysis and X-ray single crystal diffraction (Figure 1).

For optimization of the reaction conditions, various trial reactions were conducted with a combination of *N*-methylurea (**1a**), phenylacetaldehyde (**2a**) and *p*-nitrobenzaldehyde (**3a**) in order to obtain the best yield of **4a**, which is summarized in Table 1. We examined some organic solvents, and have noted that acetonitrile was the most suitable solvent among others, such as toluene, 1,4-dioxane, THF, DCE, and DCM (Table 1, entries 1–6). The catalyst loading (10%) gave the good result for the formation of the desired product (Table 1, entries 6–8).

**Table 1:** Optimization of reaction conditions.^a^

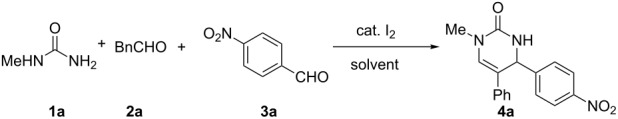					

Entry	Iodine (mol %)	Solvent	*T*	*t* (h)	Yield (%)^b^

1	10	toluene	reflux	12	56
2	10	1,4-dioxane	reflux	12	53
3	10	THF	reflux	12	58
4	10	DCE	reflux	12	52
5	10	DCM	rt	24	10
6	10	MeCN	reflux	12	70
7	15	MeCN	reflux	10	70
8	5	MeCN	reflux	24	58
9	0	MeCN	reflux	12	0

^a^All the reactions were carried out using **1a** (2.5 mmol), **2a** (2.5 mmol), and **3a** (3.75 mmol) in 3 mL solvent. ^b^Isolated yields.

The substrate scope of the molecular iodine-catalyzed one-pot three-component Biginelli-type reaction was then investigated, and the results were presented in Table 2. First, we examined the scope of the aromatic aldehydes **3**. Various aromatic aldehydes **3a**–**3l** and furfural (**3m**) were suitable substrates, and the expected products were obtained in moderate isolated yields (39–70%) (Table 2, entries 1–13). Electron-withdrawing as well as electron-donating groups on aromatic rings were tolerated, although the latter gave slightly reduced yields. It is noted that a halogen group on the aromatic ring was well tolerated to give the desired products, which can participate in subsequent transformations such as cross-coupling reactions (Table 2, entries 4–6). Furthermore, when phenylacetaldehyde (**2a**) was used instead of an aromatic aldehyde, product **4n** was isolated with good yield (81%; Table 2, entry 14). Subsequently, we investigated the scope of substituted acetaldehydes **2** (Table 2, entries 15–17). The variation of the alkyl substituent of acetaldehydes **2** is well tolerated to provide the desired products **4o**–**4q** in 48–55% isolated yields. Finally, *N*-ethylurea **1b** was also investigated in the one-pot three-component reaction, and the reactions proceeded smoothly to give the corresponding dihydropyrimidinones **4r**–**4v** in 58–72% isolated yields (Table 2, entries 18–22). Based on the experimental results above, the iodine-catalyzed Biginelli-type reaction proved to be of broad scope and provides higher yields of dihydropyrimidinones than the earlier described method with BF_3_·Et_2_O as the catalyst.

**Table 2:** One-pot synthesis of dihydropyrimidinones.^a^

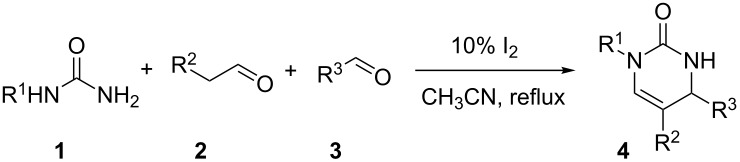					

Entry	R^1^	R^2^	R^3^	Product	Yield (%)^b^

1	Me (**1a**)	Ph (**2a**)	4-NO_2_C_6_H_4_ (**3a**)	**4a**	70
2	**1a**	**2a**	3-NO_2_C_6_H_4_ (**3b**)	**4b**	67
3	**1a**	**2a**	4-CNC_6_H_4_ (**3c**)	**4c**	68
4	**1a**	**2a**	4-ClC_6_H_4_ (**3d**)	**4d**	63
5	**1a**	**2a**	4-BrC_6_H_4_ (**3e**)	**4e**	70
6*^c^*	**1a**	**2a**	2-BrC_6_H_4_ (**3f**)	**4f**	67
7	**1a**	**2a**	4-CF_3_C_6_H_4_ (**3g**)	**4g**	56
8	**1a**	**2a**	Ph (**3h**)	**4h**	57
9	**1a**	**2a**	4-MeC_6_H_4_ (**3i**)	**4i**	53
10	**1a**	**2a**	4-MeOC_6_H_4_ (**3j**)	**4j**	46
11	**1a**	**2a**	piperonyl (**3k**)	**4k**	39
12	**1a**	**2a**	1-naphthyl (**3l**)	**4l**	49
13	**1a**	**2a**	2-furyl (**3m**)	**4m**	42
14	**1a**	**2a**	Bn (**2a**)	**4n**	81
15	**1a**	iPr (**2b**)	**3a**	**4o**	48
16	**1a**	*n*-Bu (**2c**)	**3a**	**4p**	55
17	**1a**	pentyl (**2d**)	**3a**	**4q**	54
18	Et (**1b**)	**2a**	**3a**	**4r**	72
19	Et (**1b**)	**2a**	**3b**	**4s**	66
20	Et (**1b**)	**2a**	**3c**	**4t**	67
21	Et (**1b**)	**2a**	**3d**	**4u**	58
22	Et (**1b**)	**2a**	**3e**	**4v**	63

^a^All the reactions were carried out using **1** (2.5 mmol), **2** (2.5 mmol), **3** (3.75 mmol), and iodine (0.25 mmol) in 3 mL MeCN at reflux for 12 h. ^b^Isolated yields.

**Scheme 1:**
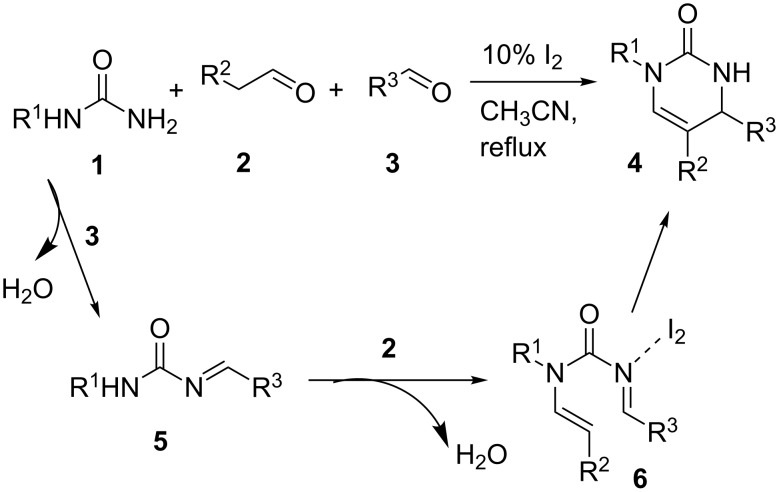
Possible mechanism.

Molecular iodine is a mild catalyst with moderate Lewis acidity. Thus, a possible mechanism was proposed in Scheme 1. The first step is the condensation via the primary nitrogen of mono-substituted urea **1** with the aromatic aldehyde **3** to give the intermediate **5**. Then, the enamide **6** is generated through the condensation of imine **5** with substituted acetaldehyde **2**. This could then undergo an iodine-catalytic intramolecular cyclisation to afford the final dihydropyrimidinone **4**.

Based on the observations above, a preliminary investigation on the catalytic asymmetric version was performed. Recently, our group has developed a novel class of spirocyclic SPINOL-phosphoric acids derived from chiral 1,1'-spirobiindane-7,7'-diol, which could effectively catalyze some highly enantioselective reactions [25–31]. These previous successes led us to envision that SPINOL-phosphoric acids would effectively catalyze the enantioselective three-component reaction of mono-substituted ureas **1**, alkylaldehydes **2** and arylaldehydes **3** to generate enantioenriched dihydropyrimidinones **4** [32–35].

**Figure 2:**
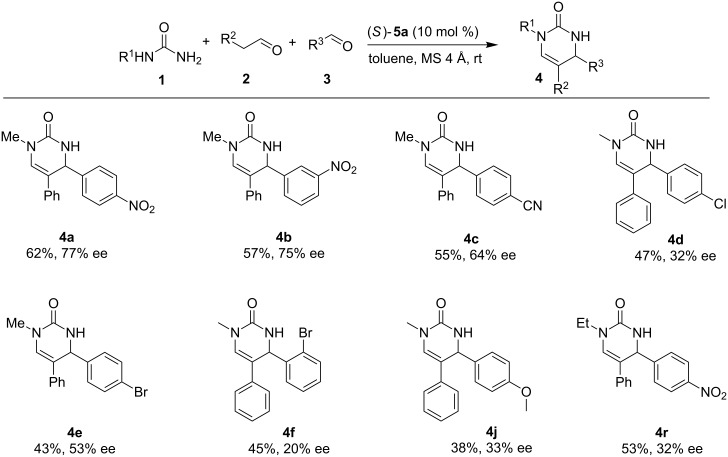
Scope of the enantioselective reaction. Reaction conditions: **5a** (10 mol %, 0.02 mmol), **1** (0.2 mmol), **2** (0.2 mmol), **3** (0.3 mmol), MS 4 Å (0.1 g), toluene (1 mL), rt, 2 days. Isolated Yields were given. The ee’s were determined by chiral HPLC.

In our initial study, we examined the multicomponent model reaction between *N*-methylurea **1a**, phenylacetaldehyde **2a**, and *p*-nitrobenzaldehyde **3a**. As shown in Table 3, optimization of the reaction conditions revealed that toluene was the best solvent, chiral SPINOL-phosphoric acid **5a** was the best catalyst and the best temperature was room temperature, which afforded product **4a** with 77% ee in 62% yield (Table 3, entry 4). With these reaction conditions identified, the variation of the reaction substrates was well tolerated to provide the desired products with up to 77% ee (Figure 2). Although the enantioselectivity was low to moderate, it should be noted that this is the first catalytic enantioselective version of this multicomponent reaction.

**Table 3:** Optimization of the asymmetric reaction conditions.^a^

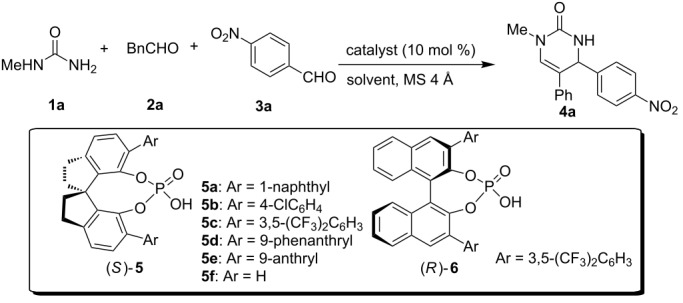					

Entry	Catalyst	Solvent	*T* (°C)	Yield (%)^b^	ee^c^

1	**5a**	CH_3_CN	rt	75	17
2	**5a**	CH_3_CN	0	51	34
3	**5a**	xylene	rt	60	72
4	**5a**	toluene	rt	62	77
5	**5a**	toluene	0	0	–
6	**5a**	toluene	50	65	67
7	**5b**	toluene	50	39	34
8	**5c**	toluene	50	30	42
9	**5d**	toluene	50	41	58
10	**5e**	toluene	50	28	60
11	**5f**	toluene	50	58	12
12	**6**	toluene	50	0	–

^a^Reaction conditions: Catalyst (10 mol %, 0.02 mmol), **1a** (0.2 mmol), **2a** (0.2 mmol), **3a** (0.3 mmol), MS 4 Å (0.1 g), solvent (1 mL), 2 days. ^b^Isolated yields. ^c^Determined by chiral HPLC analysis.

## Conclusion

In conclusion, we have demonstrated the first efficient, molecular iodine-catalyzed three-component synthesis of dihydropyrimidinones starting from simple readily available mono-substituted ureas, alkylaldehydes, and arylaldehydes. A significant progress was obtained with an extremely broad substrate scope, giving the corresponding DHPMs with reasonable yields under mild reaction conditions. Moreover, the catalytic asymmetric version of this multicomponent reaction has also been developed to a straightforward synthesis of enantiomerically enriched DHPMs by using a chiral SPINOL-phosphoric acid as the catalyst.

## Crystallographic Data

Single crystal data for compound **4a** (CCDC 918944) has been deposited in the Cambridge Crystallographic Data Centre. These data can be obtained free of charge via http://www.ccdc.cam.ac.uk/data_request/cif.

## Supporting Information

File 1 Experimental details and spectroscopic data.
