# Supplementary material for: Efficient synthesis of dihydropyrimidinones via a three-component Biginelli-type reaction of urea, alkylaldehyde and arylaldehyde
Source: Beilstein J Org Chem. 2013 Dec 11;9:2846–51. doi: 10.3762/bjoc.9.320 (PMC3869270; doi:10.3762/bjoc.9.320)
Supplement: File 1 — Experimental details and spectroscopic data. [file Beilstein_J_Org_Chem-09-2846-s001.pdf]

Supporting Information

for

Efficient synthesis of dihydropyrimidinones via a three-component Biginelli-type reaction of urea, alkylaldehyde and arylaldehyde

Haijun Qu, Xuejian Li, Fan Mo, and Xufeng Lin*

Address: Department of Chemistry, Zhejiang University, Hangzhou 310027, China

Email: Xufeng Lin - lxfok@zju.edu.cn

* Corresponding author

Experimental details and spectroscopic data

Contents

General information…………………………………………………………..S2

General procedure……………………………………………………….....S2

Characterization data…………………………………………..………..S3

NMR spectra…………………………………………………………….……..S6

HPLC spectra ………………………………………………………………..S28

General information

Melting points were obtained on a microscopical instrument and are uncorrected. NMR spectra were recorded on a 400 MHz spectrometer and TMS as internal standard. IR spectra were recorded on a FTIR spectrometer. HRMS data were obtained using EI ionization. All reagents and solvents used were commercially available. Column chromatography was carried out on silica gel column (300-400 mesh) with mixed solvents (hexane/ethyl acetate). Optical rotations were determined using a Perkin Elmer Model 341 polarimeter at 20 °C. The enantiomeric excesses (ee) were determined by chiral HPLC analysis on a Daicel Chiralpak AD-H column.

General procedure for the iodine-catalyzed synthesis of DHPMs 4

Mono-substituted urea **1** (2.5 mmol), aromatic aldehyde **3** (3.75 mmol) and iodine (0.25 mmol) were dissolved in 3 mL acetonitrile under nitrogen atmosphere. After the mixture was stirred at room temperature for 10 minutes, alkylaldehyde **2** (2.5 mmol) was added, and the resulting mixture was stirred under reflux for 12 hours. Then the reaction mixture was diluted with ethyl acetate, and washed with a solution of sodium thiosulfate followed by water. The organic phase was dried over anhydrous Na_2SO_4, and evaporation of the solvent followed by purification on silica gel afforded the pure desired DHPM **4**.

General procedure for the catalytic asymmetric synthesis of DHPMs 4.

Mono-substituted urea **1** (0.2 mmol), aromatic aldehyde **3** (0.3 mmol), MS 4 Å (0.1 g) and **5a** (0.02 mmol) were dissolved in 1 mL toluene under nitrogen atmosphere. After the mixture was stirred at room temperature for 30 minutes, alkyl aldehyde **2** (0.2 mmol) was added, and the resulting mixture was stirred at room temperature for 2 days. Then the reaction mixture was purified by flash chromatography on silica gel to afford the pure desired chiral DHPM **4**.

Characterization data

1-Methyl-4-(4-nitrophenyl)-5-phenyl-3,4-dihydropyrimidin-2(1H)-one (***4a***). Yellow solid, m.p.186-188 °C; the chiral material was determined to be of 77% ee by chiral HPLC analysis [Daicel Chiralpak AD-H, *n*-hexane / *i*-propanol = 80/20, l.0 mL/min, λ = 254 nm, t (major) = 16.60 min, t (minor) = 22.42 min]. $[\alpha]_D^{20}$ = + 5.6° (c = 0.52, $CHCl_3$). IR(neat) *v* 3250, 1682, 1651, 1519, 1497, 1455, 1403, 1270, 818, 736, 732, 700 cm^{-1}; ^1H NMR (400 MHz, $CDCl_3$) δ =8.13 (d, *J* = 8.7 Hz, 2 H), 7.46 (d, *J* = 8.7 Hz, 2 H), 7.26-7.12 (m, 5 H), 6.55 (s, 1 H), 6.14 (s, 1 H), 5.60 (d, *J* = 2.6 Hz, 1 H), 3.20 (s, 3 H) ppm. ^{13}C NMR (100 MHz, $CDCl_3$) δ= 153.24, 149.21, 147.53, 135.30, 128.78, 127.95, 127.65, 127.06, 124.86, 124.21, 112.81, 57.34, 34.79 ppm. HRMS (EI): m/z calcd for ($C_{17}H_{15}N_3O_3$): 309.1113; found: 309.1111.

1-Methyl-4-(3-nitrophenyl)-5-phenyl-3,4-dihydropyrimidin-2(1H)-one (***4b***). Yellow solid, m.p.180-182 °C; the chiral material was determined to be of 75% ee by chiral HPLC analysis [Daicel Chiralpak AD-H, *n*-hexane / *i*-propanol = 80/20, l.0 mL/min, λ = 254 nm, t (major) = 14.72 min, t (minor) = 21.31 min]. $[\alpha]_D^{20}$ = +8.2° (c = 0.46, $CHCl_3$). IR（neat）*v* 3239, 3084, 2928, 1681, 1599, 1497, 1456, 1404, 1349, 1270, 759, 737, 693, 596 cm^{-1}; ^1H NMR (400 MHz, $CDCl_3$) δ =8.16 (s, 1 H), 8.08 (d, *J* = 8.2 Hz, 1 H), 7.63 (d, *J* = 7.7 Hz, 1 H), 7.45 (t, *J* = 7.9 Hz, 1 H), 7.26 (s, 1 H), 7.22 (s, 1 H), 7.16 (t, *J* = 6.6 Hz, 3 H), 6.63 (s, 1 H), 6.28 (s, 1 H), 5.60 (d, *J* = 2.5 Hz, 1 H), 3.23 (s, 3 H) ppm. ^{13}C NMR (100 MHz, $CDCl_3$) δ= 153.41, 148.48, 144.36, 135.27, 132.8 , 129.84, 128.76, 128.22, 126.93, 124.63, 122.98, 122.02, 112.39, 56.96, 34.79 ppm. HRMS (EI): m/z calcd for ($C_{17}H_{15}N_3O_3$): 309.1113; found: 309.1112.

4-(1-Methyl-2-oxo-5-phenyl-1,2,3,4-tetrahydropyrimidin-4-yl)benzonitrile (***4c***). White solid, m.p. 113-115 °C; the chiral material was determined to be of 64% ee by chiral HPLC analysis [Daicel Chiralpak AD-H, *n*-hexane / *i*-propanol = 80/20, l.0 mL/min, λ = 254 nm, t (major) = 16.34 min, t (minor) = 26.66 min]. $[\alpha]_D^{20}$ = +4.8° (c = 0.41, $CHCl_3$); IR(neat) *v* 3249, 2925, 2228, 1679, 1603, 1498, 1456, 1404, 1327, 1269, 1108, 765, 731, 694, 587 cm^{-1}; ^1H NMR (400 MHz, $CDCl_3$) δ 7.55 (d, *J* = 7.2 Hz, 2 H), 7.38 (d, *J* = 7.9 Hz, 2 H), 7.26-7.19 (m, 2 H), 7.14 (dd, *J* = 17.6, 7.4 Hz, 3 H), 6.53 (s, 1 H), 6.08 (br, 1 H), 5.52 (s, 1 H), 3.17 (s, 3 H) ppm. ^{13}C NMR (100 MHz, $CDCl_3$) δ 153.26, 147.33, 135.37, 132.72, 128.72, 127.90, 127.47, 126.96, 124.81, 118.44, 112.77, 111.83, 57.50, 34.72 ppm. HRMS (EI): m/z calcd for ($C_{18}H_{15}N_3O$): 289.1215; found: 289.1225.

4-(4-Chlorophenyl)-1-methyl-5-phenyl-3,4-dihydropyrimidin-2(1H)-one (***4d***). White solid ,m.p: 149-151℃; the chiral material was determined to be of 32% ee by chiral HPLC analysis [Daicel Chiralpak AD-H, *n*-hexane / *i*-propanol =90/10,0.8 mL/min, λ = 254 nm, t (major) = 29.13min, t (minor) = 31.84 min]. IR (neat) *v* 3108, 1671, 1647, 1593, 1526, 1491, 1462, 1401, 1328, 726, 700 cm^{-1}; ^1H NMR (400 MHz, $CDCl_3$) δ= 7.21 (m, 9 H), 6.53 (s, 1 H), 5.51 (s, 1 H), 5.44 (d, *J* = 2.1 Hz, 1 H), 3.19 (s, 3 H) ppm. ^{13}C NMR (100 MHz, $CDCl_3$) δ=140.88, 135.67, 133.68, 129.02, 128.57, 128.13, 127.48, 126.70, 124.81, 124.81, 113.30, 57.32, 34.69 ppm. HRMS (EI): m/z calcd for ($C_{17}H_{15}ClN_2O$): 298.0873; found: 298.0870.

4-(4-Bromophenyl)-1-methyl-5-phenyl-3,4-dihydropyrimidin-2(1H)-one (***4e***). White solid, m.p. 170-172 °C; the chiral material was determined to be of 53% ee by chiral HPLC analysis [Daicel Chiralpak AD-H, *n*-hexane / *i*-propanol = 80/20, l.0 mL/min, λ = 254 nm, t (major) = 13.68 min, t (minor) = 18.87 min]. $[\alpha]_D^{20}$ = +3° (c = 0.44, $CHCl_3$). IR(neat) *v* 3265, 1673, 1485, 1455, 1405, 1325, 1266, 1003, 754, 730, 694 cm^{-1}; ^1H NMR (400 MHz, $CDCl_3$) δ =7.41 (d, J = 8.3 Hz, 2 H), 7.25-7.19 (m, 2 H), 7.15 (t, J = 7.8 Hz, 5 H), 6.52 (s, 1 H), 5.56 (s, 1 H), 5.43 (d, J = 2.1 Hz, 1 H), 3.19 (s, 3 H) ppm. ^{13}C NMR (100 MHz, $CDCl_3$) δ =153.29, 141.39, 135.71, 132.00, 128.59, 128.47, 127.51, 126.75, 124.85, 121.93, 113.30, 57.46, 34.69 ppm. HRMS (EI): m/z calcd for ($C_{17}H_{15}BrN_2O$): 342.0368; found: 340.0211.

4-(2-Bromophenyl)-1-methyl-5-phenyl-3,4-dihydropyrimidin-2(1H)-one (***4f***). White solid, m.p: 173-175℃; the chiral material was determined to be of 20% ee by chiral HPLC analysis [Daicel Chiralpak AD-H, *n*-hexane / *i*-propanol = 90/10, 0.8 mL/min, λ = 254 nm, t (major) = 29.30 min, t (minor) = 31.37min]. IR (neat) *v* 3242, 3080, 2923, 2238, 1678, 1597, 1496, 1470, 1402, 1325, 1262, 1185, 1111, 757, 730, 695, 599 cm^{-1}; ^1H NMR (400 MHz, $CDCl_3$) δ =7.46 (s, 1 H), 7.39 (d, J = 7.8 Hz, 1 H), 7.23 (m, 3 H), 7.17 (m, 4 H), 6.58 (s, 1 H), 5.52 (s, 1 H), 5.43 (d, J = 2.3 Hz, 1 H), 3.23 (s, 3 H) ppm. ^{13}C NMR (100 MHz, $CDCl_3$) δ=153.14, 144.57, 135.65, 131.17, 130.51, 129.87, 128.60, 127.78, 126.73, 125.41, 124.77, 122.99, 112.83, 57.58, 34.76 ppm. HRMS (EI): m/z calcd for ($C_{17}H_{15}BrN_2O$): 342.0368; found: 340.0207.

1-Methyl-5-phenyl-4-(4-(trifluoromethyl)phenyl)-3,4-dihydropyrimidin-2(1H)-one (***4g***). White solid, m.p. 130-132 °C; IR(neat) *v* 3318, 1663, 1598, 1496, 1451, 1406, 1324, 1167, 1125, 1067, 1009, 855, 730, 691 cm^{-1}; ^1H NMR (400 MHz, $CDCl_3$) δ= 7.55 (d, *J* = 8.2 Hz, 2 H), 7.41 (d, *J* = 8.1 Hz, 2 H), 7.26-7.21 (m, 2 H), 7.17 (t, *J* = 6.7 Hz, 3 H), 6.56 (s, 1 H), 5.67 (s, 1 H), 5.54 (d, *J* = 2.4 Hz, 1 H), 3.21 (s, 3 H) ppm. ^{13}C NMR (100 MHz, $CDCl_3$) δ =153.31, 146.15, 135.61, 130.20 (*J*=33 Hz), 128.70, 127.77, 126.88, 125.98 (*J*=229 Hz), 125.97, 125.93, 125.27, 113.14, 57.59, 34.74 ppm. HRMS (EI): m/z calcd for ($C_{18}H_{15}F_3N_2O$): 332.1136; found: 332.1138.

*1-Methyl-4,5-diphenyl-3,4-dihydropyrimidin-2(1H)-one (**4h**).* White solid, m.p. 179-181 °C; IR (neat) *v* 3319, 1681, 1651, 1599, 1495, 1454, 1403, 1326, 1269, 754, 732, 697, 595 cm^{-1}: ^1H NMR (400 MHz, $CDCl_3$) δ = 7.33-7.14 (m, 10 H), 6.55 (s, 1 H), 5.44 (d, J = 2.2 Hz, 1 H), 5.31 (s, 1 H), 3.20 (s, 3 H) ppm. ^{13}C NMR (100 MHz, $CDCl_3$) δ = 153.26, 142.37, 136.05, 128.93, 128.48, 128.04, 127.38, 126.70, 126.55, 124.88, 113.60, 58.22, 34.68 ppm. HRMS (EI): m/z calcd for ($C_{17}H_{16}N_2O$): 264.1263; found: 264.1264.

*Methyl-5-phenyl-4-(p-tolyl)-3,4-dihydropyrimidin-2(1H)-one (**4i**).* White solid, m.p. 158-160 °C; IR (neat) *v* 3247, 3058, 2920, 1678, 1496, 1455, 1402, 1328, 1271, 1105, 1048, 751, 682, 593 cm^{-1}; ^1H NMR (400 MHz, $CDCl_3$) δ =7.24-7.05 (m, 9 H), 6.53 (s, 1 H), 5.40 (d, *J* = 2.0 Hz, 1 H), 5.29 (s, 1 H), 3.19 (s, 3 H), 2.28 (s, 3 H) ppm. ^{13}C NMR (100 MHz, $CDCl_3$) δ= 153.29, 139.50, 137.73, 136.13, 129.58, 128.45, 127.26, 126.60, 126.48, 124.84, 113.71, 57.89, 34.66, 21.04 ppm. HRMS (EI): m/z calcd for ($C_{17}H_{18}N_2O_2$): 278.1419; found: 278.1418.

*4-(4-Methoxyphenyl)-1-methyl-5-phenyl-3,4-dihydropyrimidin-2(1H)-one (**4j**).* White solid,m.p: 168-170°C;the chiral material was determined to be of 33% ee by chiral HPLC analysis [Daicel Chiralpak AD-H, *n*-hexane / *i*-propanol = 90/10, 0.8 mL/min, λ = 254 nm, t (major) = 43.37min, t (minor) = 48.50min]; IR (neat) *v* 3248, 1681, 1607, 1509, 1455, 1403, 1326, 1251, 1174, 752 cm^{-1}; ^1H NMR (400 MHz, $CDCl_3$) δ 7.25-7.19 (m, 4 H), 7.16 (m, 3 H), 6.81 (d, *J* = 8.6 Hz, 2 H), 6.53 (s, 1 H), 5.39 (d, *J* = 2.1 Hz, 1 H), 5.27 (s, 1 H), 3.76 (s, 3 H), 3.20 (s, 3 H) ppm. ^{13}C NMR (100 MHz, $CDCl_3$) δ 159.30, 153.21, 136.13, 134.71, 128.46, 127.94, 127.19, 126.51, 124.87, 114.25, 113.81, 57.68, 55.17, 34.67 ppm. HRMS(EI): m/z calcd for ($C_{18}H_{18}N_2O_2$): 294.1368; found: 294.1369.

*4-(Benzo[d][1,3]dioxol-5-yl)-1-methyl-5-phenyl-3,4-dihydropyrimidin-2(1H)-one (**4k**).* White solid, m.p. 202-204 °C; IR(neat) *v* 3319, 2917, 1675, 1590, 1499, 1486, 1399, 1334, 1246, 1108, 1033, 685, 593 cm^{-1}; ^1H NMR (400 MHz, $CDCl_3$) δ 7.27-7.13 (m, 5 H), 6.84-6.68 (m, 3 H), 6.56 (s, 1 H), 5.92 (s, 2 H), 5.37 (d, *J* = 1.9 Hz, 1 H), 5.28 (s, 1 H), 3.22 (s, 3 H) ppm. ^{13}C NMR (100 MHz, $CDCl_3$) δ 153.13, 148.21, 147.38, 136.54, 135.95, 128.50, 127.35, 126.58, 124.79, 120.19, 113.52, 108.35, 107.11, 101.08), 58.00, 34.69 (s) ppm. HRMS (EI): m/z calcd for ($C_{18}H_{16}N_2O_3$): 308.1161; found: 308.1165.

*1-Methyl-4-(naphthalen-1-yl)-5-phenyl-3,4-dihydropyrimidin-2(1H)-one (**4l**).* White solid, m.p. 162-164 °C; IR (neat) *v* 3238, 3059, 2925, 1678, 1598, 1497, 1455, 1403, 1327, 1266, 95, 780, 731, 694 $^{-1}$cm ; ^1H NMR (400 MHz, $CDCl_3$) δ 8.14 (d, *J* = 8.4 Hz, 1 H), 7.91 (d, *J* = 8.1 Hz, 1 H), 7.79 (d, *J* = 8.2 Hz, 1 H), 7.61 (t, *J* = 7.6 Hz, 1 H), 7.53 (m, 2H), 7.38 (t, *J* = 7.7 Hz, 1 H), 7.18 – 7.07 (m, 5 H), 6.84 (s, 1 H), 6.28 (d, *J* = 1.7 Hz, 1 H), 5.34 (s, 1 H), 3.23 (s, 3 H) ppm. ^{13}C NMR (100 MHz, $CDCl_3$) δ 153.17, 135.84, 135.79, 134.41, 130.25, 129.37, 128.83, 128.49, 128.40, 126.92, 126.48, 125.78, 125.09, 124.37, 121.77, 112.11, 53.62, 34.76 ppm. HRMS (EI): m/z calcd for ($C_{21}H_{18}N_2O$): 314.1419; found: 314.1419.

*4-(Furan-2-yl)-1-methyl-5-phenyl-3,4-dihydropyrimidin-2(1H)-one (**4m**).* White solid, m.p. 161-163 °C; IR (neat) *v* 3241, 2921, 1678, 1599, 1497, 1455, 1403, 1326, 1250, 1108, 1000, 780, 748, 694, 593 cm^{-1}; ^1H NMR (400 MHz, $CDCl_3$) δ 7.36 (s, 1 H), 7.30 (d, *J* = 7.8 Hz, 1 H), 7.26 (t, *J* = 5.9 Hz, 3 H), 7.20 (t, *J* = 6.9 Hz, 1 H), 6.61 (s, 1 H), 6.27 (s, 1 H), 6.16 (d, *J* = 2.5 Hz, 1 H), 5.48 (s, 1 H), 5.39 (s, 1 H), 3.21 (s, 3 H) ppm . ^{13}C NMR (100 MHz, $CDCl_3$) δ 154.17, 153.66, 142.55, 135.61, 128.60, 128.09, 126.66, 124.35, 111.13, 110.38, 106.94, 51.08, 34.77 ppm. HRMS (EI): m/z calcd for ($C_{15}H_{14}N_2O_2$): 254.1055; found: 254.1051.

*4-Benzyl-1-methyl-5-phenyl-3,4-dihydropyrimidin-2(1H)-one (**4n**).* White solid, m.p. 91-93 °C; IR (neat) *v* 3238, 1681, 1599, 1495, 1454, 1408, 132, 1269,752,698 cm^{-1}; ^1H NMR (400 MHz, $CDCl_3$) δ 7.40-7.33 (m, 4H), 7.31 (t, *J* = 7.3 Hz, 2 H), 7.25 (t, *J* = 6.9 Hz, 2 H), 7.17 (d, J = 7.3 Hz, 2 H), 6.32 (s, 1 H), 5.15 (s, 1 H), 4.66 (d, *J* = 8.4 Hz, 1 H), 3.00 (s, 3 H), 2.90 (m, 1 H), 2.77-2.66 (m, 1 H) ppm. ^{13}C NMR (100 MHz, $CDCl_3$) δ 153.97, 136.63, 135.85, 129.64, 128.88, 128.44, 127.16, 126.72, 126.67, 124.50, 114.11, 55.21, 42.41, 34.37 ppm ; HRMS (EI): m/z calcd for ($C_{18}H_{18}N_2O$): 278.1419; found: 278.1418.

*5-Isopropyl-1-methyl-4-(4-nitrophenyl)-3,4-dihydropyrimidin-2(1H)-one (**4o**).* Yellow liquid, IR (neat) *v* 3249, 2940, 1678, 1521, 1462, 1341, 1262, 1113, 858, 688 cm^{-1}; ^1H NMR (400 MHz, $CDCl_3$) δ 8.20 (d, *J* = 8.6 Hz, 2 H), 7.46 (d, *J* = 8.6 Hz, 2 H), 5.86 (s, 1 H), 5.56 (s, 1 H), 5.08 (d, *J* = 1.9 Hz, 1 H), 3.10 (s, 3 H), 1.95 (m, 1 H), 1.00 (d, *J* = 6.8 Hz, 3 H), 0.95 (d, *J* = 7.0 Hz, 3 H) ppm. ^{13}C NMR (100 MHz, $CDCl_3$) δ 153.20, 150.19, 147.67, 127.87, 124.28, 124.16, 119.07, 58.06, 34.45, 28.43, 21.98, 20.73 ppm. HRMS (EI): m/z calcd for ($C_{14}H_{17}N_3O_3$): 275.1270; found: 275.1269.

*5-Butyl-1-methyl-4-(4-nitrophenyl)-3,4-dihydropyrimidin-2(1H)-one (**4p**).* Yellow solid, m.p. 121-123 °C; IR (neat) *v* 3258, 2956, 2929, 2869, 1673, 1596, 1520, 1465, 1396, 1346, 1310, 1268, 1108, 1036, 855, 754, 697 cm^{-1}; ^1H NMR (400 MHz, $CDCl_3$) δ 8.19 (d, *J* = 8.6 Hz, 2 H), 7.45 (d, *J* = 8.6 Hz, 2 H), 5.80 (s, 1 H), 5.37 (s, 1 H), 5.02 (s, 1 H), 3.07 (s, 3 H), 1.72 (d, *J* = 4.5 Hz, 2 H), 1.35-1.18 (m, 4 H), 0.83 (t, *J* = 6.9 Hz, 3 H) ppm. ^{13}C NMR (100 MHz, $CDCl_3$) δ 153.20, 149.87, 147.61, 127.81, 125.17, 124.05, 112.71, 58.95, 34.27, 29.88, 29.14, 22.10, 13.74 ppm. HRMS (EI): m/z calcd for ($C_{15}H_{19}N_3O_3$): 289.1426; found: 289.1421.

*1-Methyl-4-(4-nitrophenyl)-5-pentyl-3,4-dihydropyrimidin-2(1H)-one (**4q**).* Yellow solid, m.p. 127-129 °C; IR (neat) *v* 3259, 295, 2867, 1674, 1520, 1466, 1346, 1268, 1110, 854, 753, 697 cm^{-1}; ^1H NMR (400 MHz,

$CDCl_3$) δ 8.21 (d, *J* = 8.6 Hz, 2 H), 7.47 (d, *J* = 8.6 Hz, 2 H), 5.83 (s, 1 H), 5.31 (s, 1 H), 5.04 (s, 1 H), 3.09 (s, 3 H), 1.72 (t, 2 H), 1.32-1.15 (m, 6 H), 0.86 (t, *J* = 6.8 Hz, 3 H) ppm. ^{13}C NMR (100 MHz, $CDCl_3$) δ 153.13, 149.85, 147.67, 127.83, 125.19, 124.09, 112.76, 59.07, 34.31, 31.21, 30.18, 26.70, 22.34, 13.90 ppm. HRMS (EI): m/z calcd for ($C_{16}H_{21}N_3O_3$): 303.1583; found: 303.1583.

*1-Ethyl-4-(4-nitrophenyl)-5-phenyl-3,4-dihydropyrimidin-2(1H)-one (**4r**).* Yellow solid, m.p. 182-184 ^{o}C; the chiral material was determined to be of 32% ee by chiral HPLC analysis [Daicel Chiralpak AD-H, *n*-hexane / *i*-propanol = 80/20, 0.8 mL/min, λ = 254 nm, t (major) = 28.16 min, t (minor) = 37.97 min]. $[\alpha]_D^{20}$ = +14.8°; (c = 0.36,$CHCl_3$); IR (neat) *v* 3299, 2932, 1678, 1598, 1520, 1497, 1453, 1347, 1271, 1236, 1121, 817, 757, 731, 699 cm^{-1}; ^{1}H NMR (400 MHz, $CDCl_3$) δ 8.14 (d, *J* = 8.4 Hz, 2 H), 7.46 (d, *J* = 8.4 Hz, 2 H), 7.24 (d, *J* = 7.4 Hz, 2 H), 7.17 (m, 3 H), 6.58 (s, 1 H), 5.97 (s, 1 H), 5.58 (s, 1 H), 3.63 (m, 2 H), 1.27 (t, *J* = 7.1 Hz, 3 H) ppm. ^{13}C NMR (100 MHz, $CDCl_3$) δ 152.75, 149.32, 147.49, 135.46, 128.75, 127.61, 126.99, 126.53, 124.80, 113.05, 57.12, 42.17, 14.43 ppm. HRMS (EI): m/z calcd for ($C_{18}H_{17}N_3O_3$): 323.1270; found: 323.1270.

*1-Ethyl-4-(3-nitrophenyl)-5-phenyl-3,4-dihydropyrimidin-2(1H)-one (**4s**).* Light yellow solid, m.p. 150-152 ^{o}C; IR (neat) *v* 3226, 3083, 2932, 1678, 1599, 1529, 1497, 1453, 1424, 1349, 1269, 1235, 1122, 1083, 909, 803, 758, 736, 693, 593 cm^{-1}; ^{1}H NMR (400 MHz, $CDCl_3$) δ 8.17 (s, 1 H), 8.09 (d, *J* = 8.2 Hz, 1 H), 7.63 (d, *J* = 7.7 Hz, 1 H), 7.30-7.20 (m, 1 H), 7.23 (d, *J* = 7.2 Hz, 2 H), 7.21-7.12 (m, 3 H), 6.66 (s, 1 H), 6.11 (s, 1 H), 5.57 (d, *J* = 2.5 Hz, 1 H), 3.78-3.53 (m, 2 H), 1.29 (t, *J* = 7.1 Hz, 3 H) ppm. ^{13}C NMR (101 MHz, $CDCl_3$) δ 152.72, 148.57, 144.47, 135.41, 132.86, 129.91, 128.76, 126.93, 126.76, 124.64, 123.02, 121.91, 112.88, 77.32, 77.00, 76.68, 57.01, 42.23, 14.45 ppm. HRMS (EI): m/z calcd for ($C_{18}H_{17}N_3O_3$): 323.1270; found: 323.1273.

*4-(1-Ethyl-2-oxo-5-phenyl-1,2,3,4-tetrahydropyrimidin-4-yl)benzonitrile (**4t**).* White solid, m.p. 209-211 ^{o}C; IR (neat) *v* 3241, 3083, 2976, 2933, 2228, 1681, 1603, 1498, 1454, 1372, 1270, 1236, 1180, 1122, 909, 851, 803, 765, 732, 695, 592, 554 cm^{-1}; ^{1}H NMR (400 MHz, $CDCl_3$) δ 7.59 (d, *J* = 7.8 Hz, 2 H), 7.40 (d, *J* = 7.9 Hz, 2 H), 7.24 (d, *J* = 7.7 Hz, 2 H), 7.17 (m, 3 H), 6.57 (s, 1 H), 5.52 (s, 1 H), 5.46 (s, 1 H), 3.73 – 3.54 (m, 2 H), 1.27 (t, *J* = 6.7 Hz, 3 H) ppm. ^{13}C NMR (100 MHz, $CDCl_3$) δ 152.76, 147.41, 135.49, 132.70, 128.69, 127.44, 126.90, 126.46, 124.74, 118.45, 112.99, 111.75, 57.30, 42.11, 14.39 ppm. HRMS (EI): m/z calcd for ($C_{19}H_{17}N_3O$): 303.1372; found: 303.1370.

*4-(4-Chlorophenyl)-1-ethyl-5-phenyl-3,4-dihydropyrimidin-2(1H)-one (**4u**).* White solid, m.p. 191-193 ^{o}C; IR (neat) *v* 3223, 3082, 2931, 1673, 1598, 1485, 1452, 1427, 1370, 1267, 1235, 1121, 1071, 1010, 907, 801, 756, 731, 693, 593 cm^{-1}; ^{1}H NMR (400 MHz, $CDCl_3$) δ 7.25 (m, 5 H), 7.22- 7.13 (m, 4 H), 6.56 (s, 1 H), 5.76 (s, 1 H), 5.42 (s, 1 H), 3.61 (m,, 2 H), 1.26 (t, *J* = 7.1 Hz, 3 H) ppm. ^{13}C NMR (100 MHz, $CDCl_3$) δ 152.84, 140.95, 135.88, 133.74, 129.07, 128.58, 128.10, 126.72, 126.05, 124.82, 113.66, 57.26, 42.09, 14.43 ppm. HRMS (EI): m/z calcd for ($C_{18}H_{17}ClN_2O$): 312.1029; found: 312.1029.

*4-(4-Bromophenyl)-1-ethyl-5-phenyl-3,4-dihydropyrimidin-2(1H)-one (**4v**).* White solid, m.p. 189-191 ^{o}C; IR(neat) *v* 3227, 2973, 1676, 1597, 1489, 1452, 1371, 1267, 1235, 1121, 1089, 1014, 907, 802, 757, 730, 694, 594 cm^{-1}; ^{1}H NMR (400 MHz, $CDCl_3$) δ 7.40 (d, *J* = 8.3 Hz, 2 H), 7.25-7.20 (m, 2 H), 7.15 (m,5 H), 5.63 (s, 1 H), 5.40 (d, *J* = 2.3 Hz, 1 H), 3.74 -3.51 (m, 2 H), 1.26 (t, *J* = 7.1 Hz, 3 H) ppm. ^{13}C NMR (100 MHz, $CDCl_3$) δ 152.66, 141.50, 135.90, 132.02, 128.58, 128.44, 126.70, 126.13, 124.81, 121.90, 113.50, 57.37, 42.08, 14.44 ppm. HRMS (EI): m/z calcd for ($C_{18}H_{17}BrN_2O$): 356.0524; found: 356.0524.

NMR spectra for compounds 4a-v

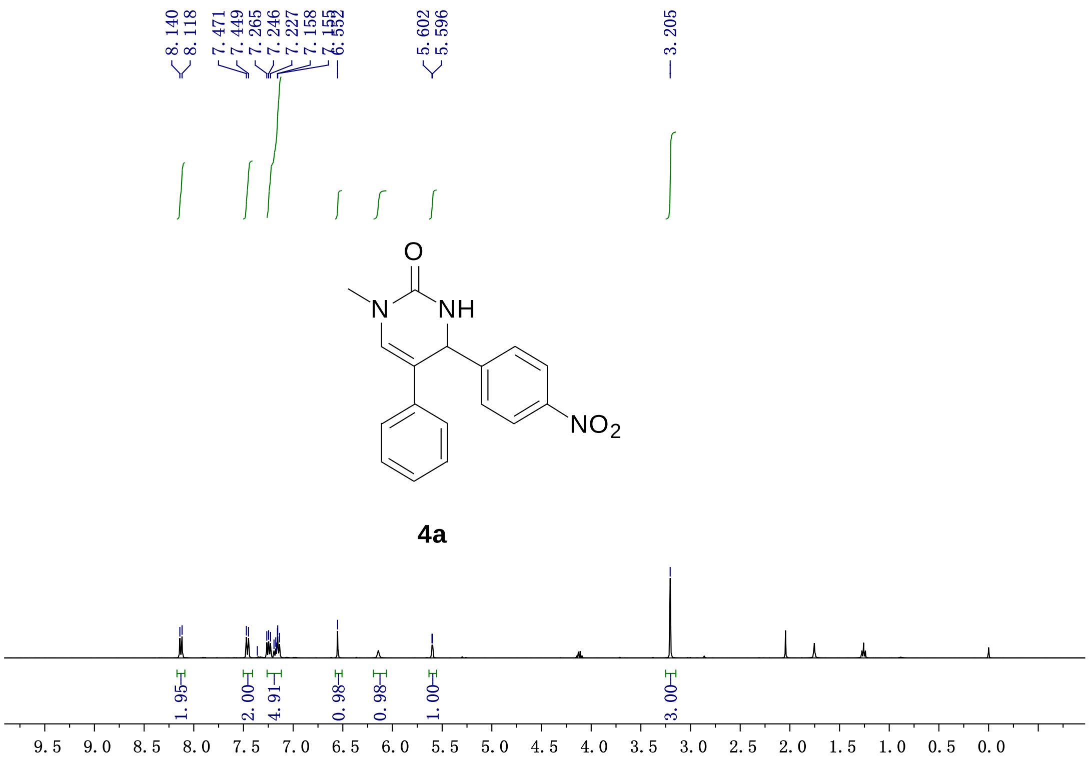

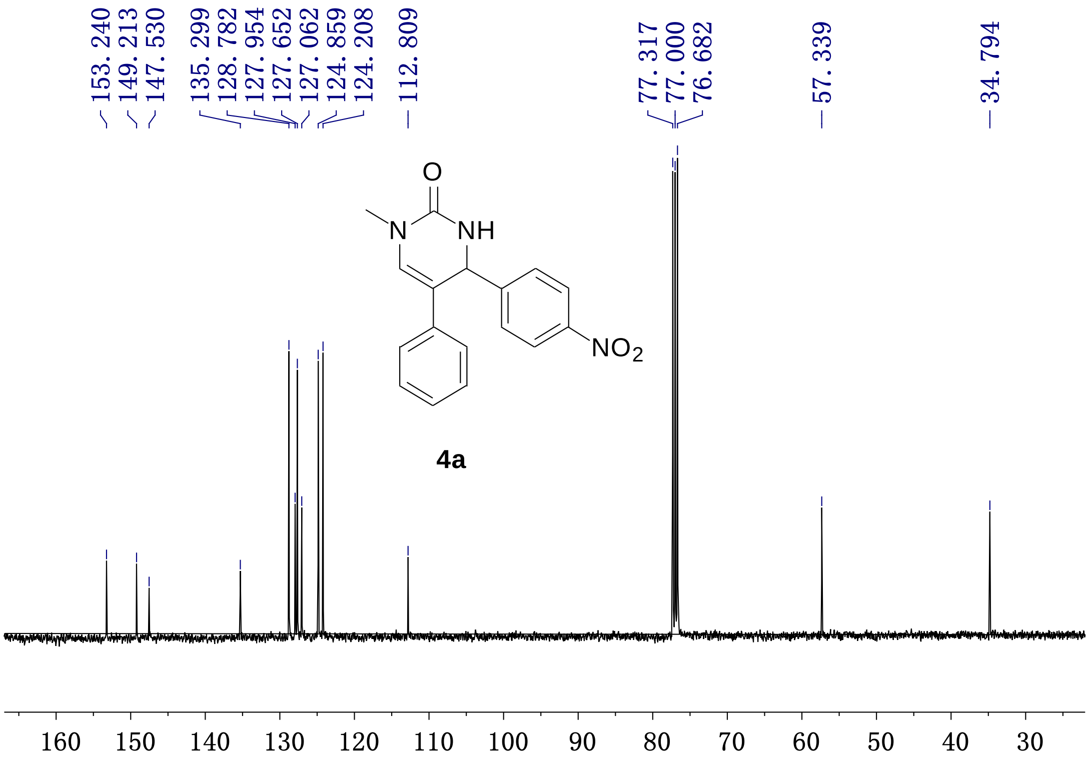

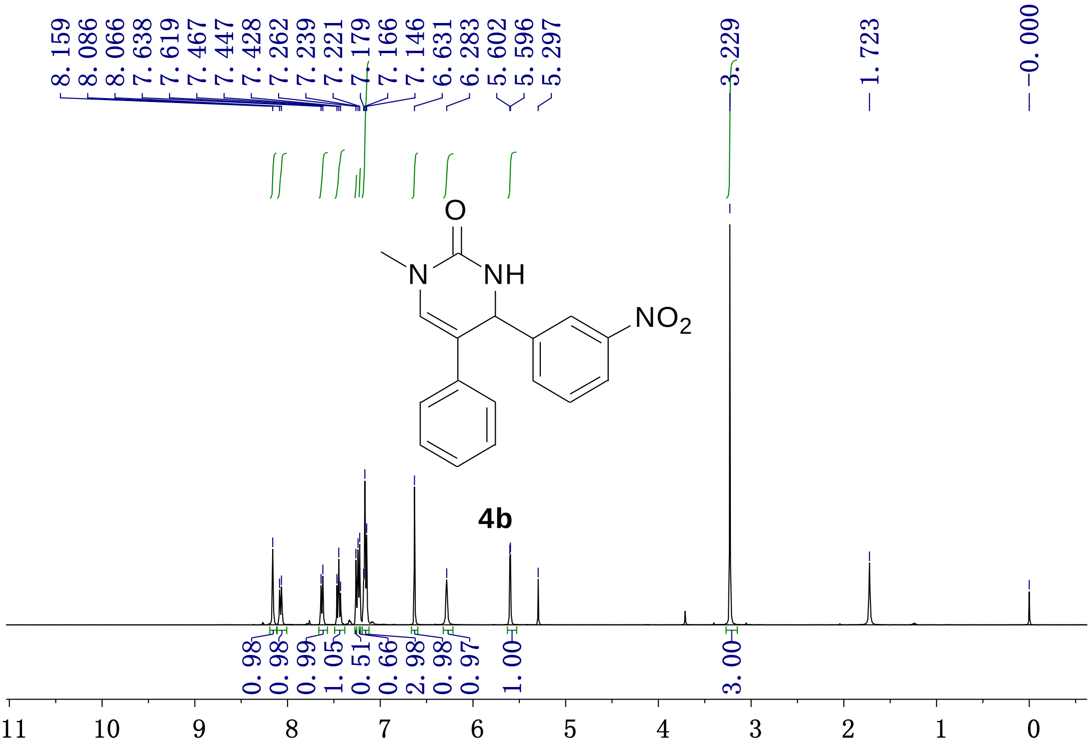
8.159
8.086
8.066
7.638
7.619
7.467
7.447
7.428
7.262
7.239
7.221
7.179
7.166
7.146
6.631
6.283
5.602
5.596
5.297
3.229
1.723
-0.000
O
N
NH
NO2
4b
0.98
0.98
0.99
1.05
0.51
0.66
2.98
0.98
0.97
1.00
3.00
11
10
9
8
7
6
5
4
3
2
1
0

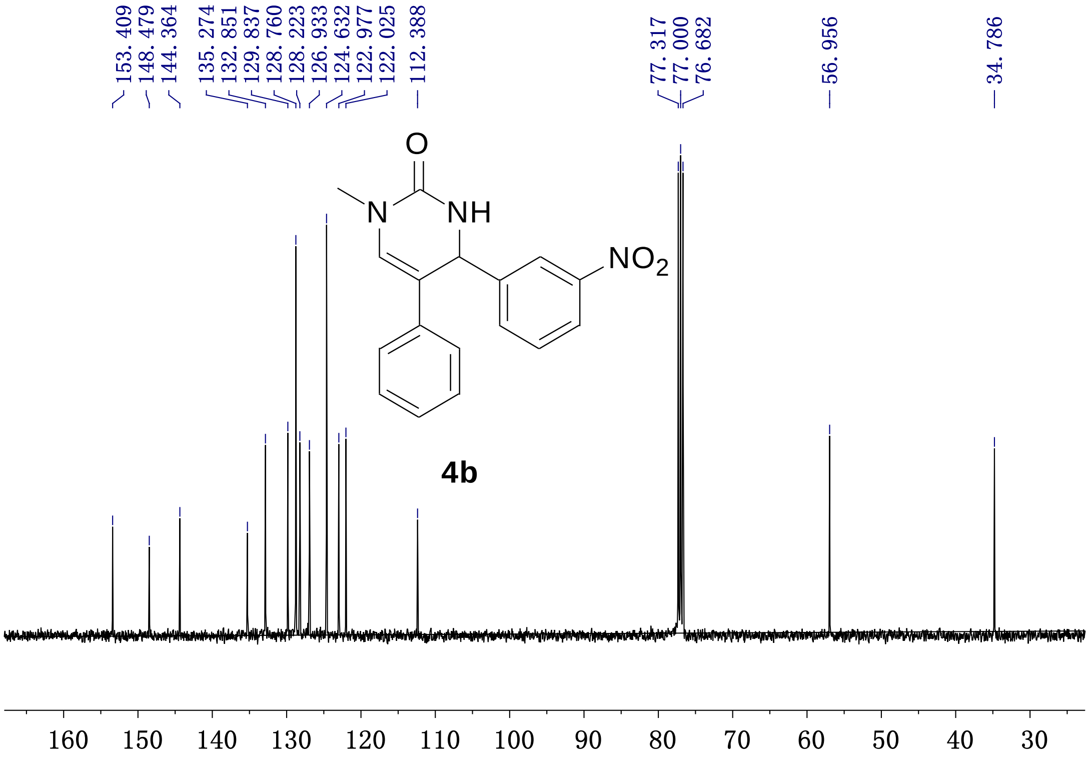
153.409
148.479
144.364
135.274
132.851
129.837
128.760
128.223
126.933
124.632
122.977
122.025
112.388
77.317
77.000
76.682
56.956
34.786
O
N
NH
NO2
4b
160
150
140
130
120
110
100
90
80
70
60
50
40
30

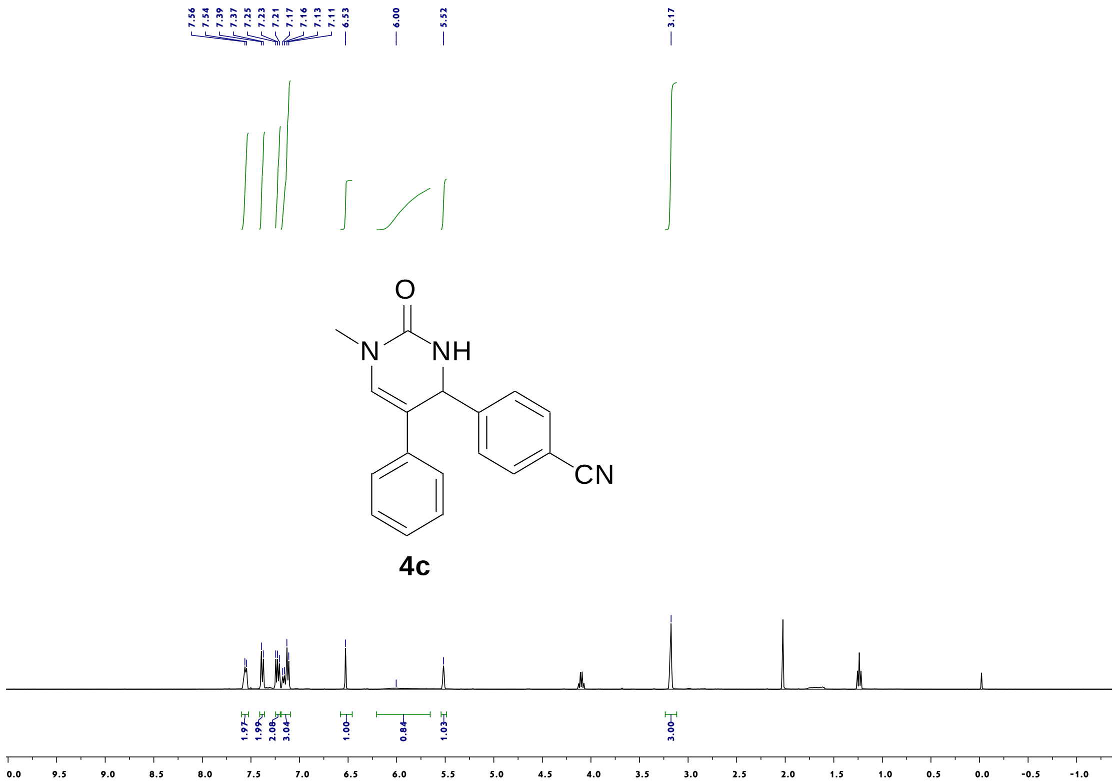
7.56
7.54
7.39
7.37
7.25
7.23
7.21
7.17
7.16
7.13
7.11
6.53
6.00
5.52
3.17
O
N
NH
CN
1.97
1.99
2.06
3.04
1.00
0.84
1.03
3.00

4c

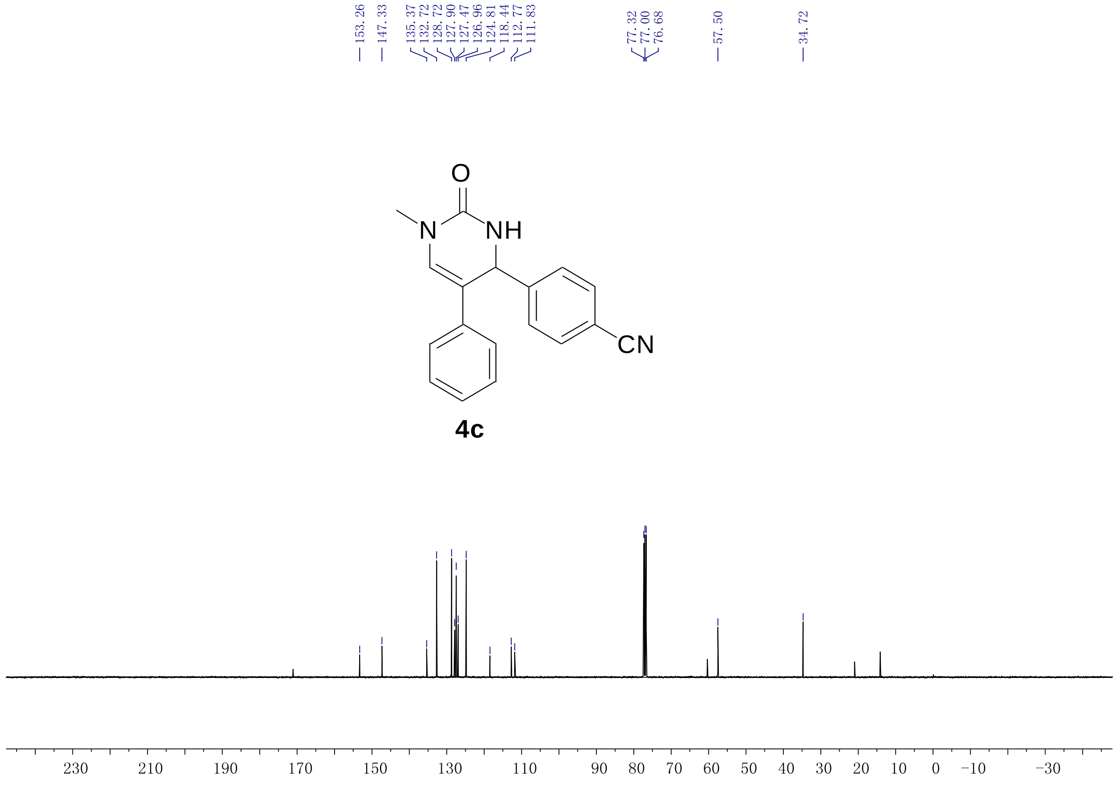
153.26
147.33
135.37
132.72
128.72
127.90
127.47
126.96
124.81
118.44
112.77
111.83
77.32
77.00
76.68
57.50
34.72
O
N
NH
CN

4c

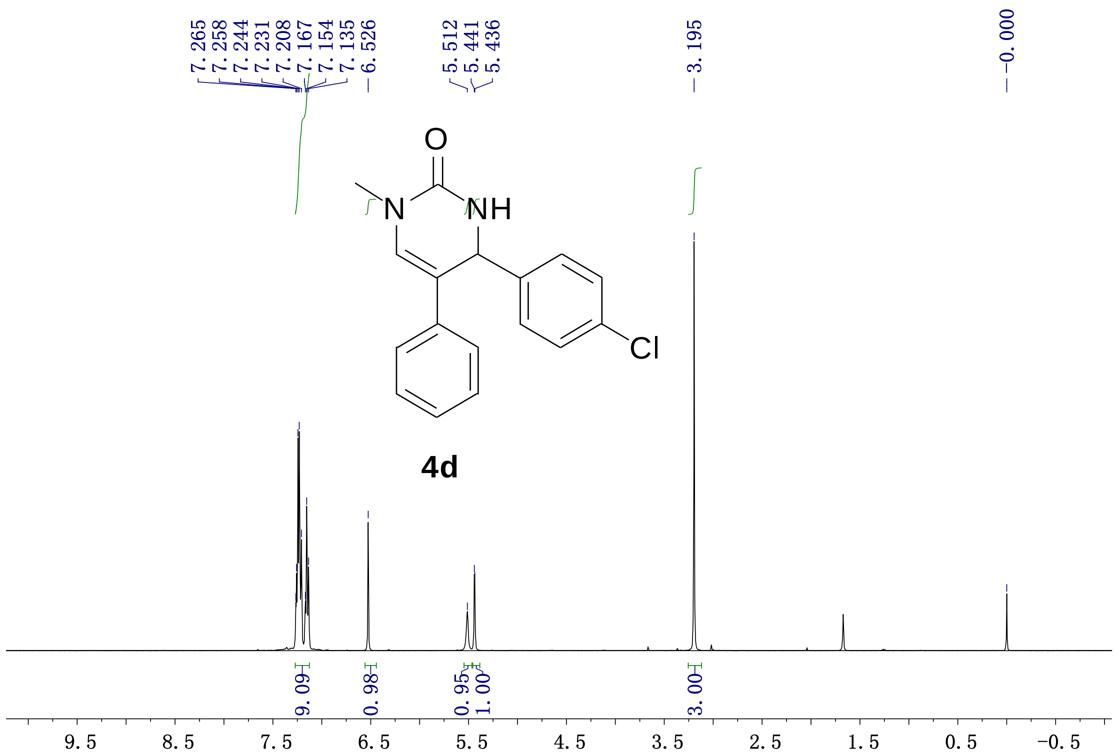
7.265
7.258
7.244
7.231
7.208
7.167
7.154
7.135
6.526
5.512
5.441
5.436
3.195
-0.000
O
N
NH
Cl
4d
9.09
0.98
0.95
1.00
3.00
9.5
8.5
7.5
6.5
5.5
4.5
3.5
2.5
1.5
0.5
-0.5

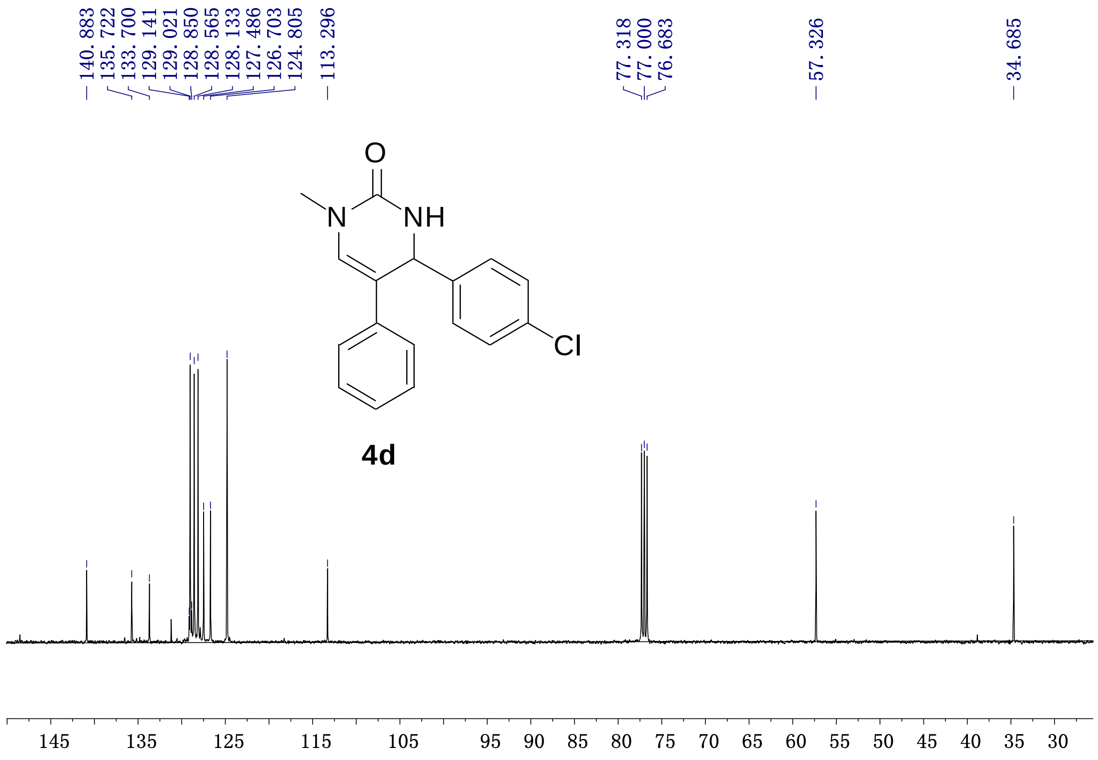
140.883
135.722
133.700
129.141
129.021
128.850
128.565
128.133
127.486
126.703
124.805
113.296
77.318
77.000
76.683
57.326
34.685
O
N
NH
Cl
4d
145
135
125
115
105
95
90
85
80
75
70
65
60
55
50
45
40
35
30

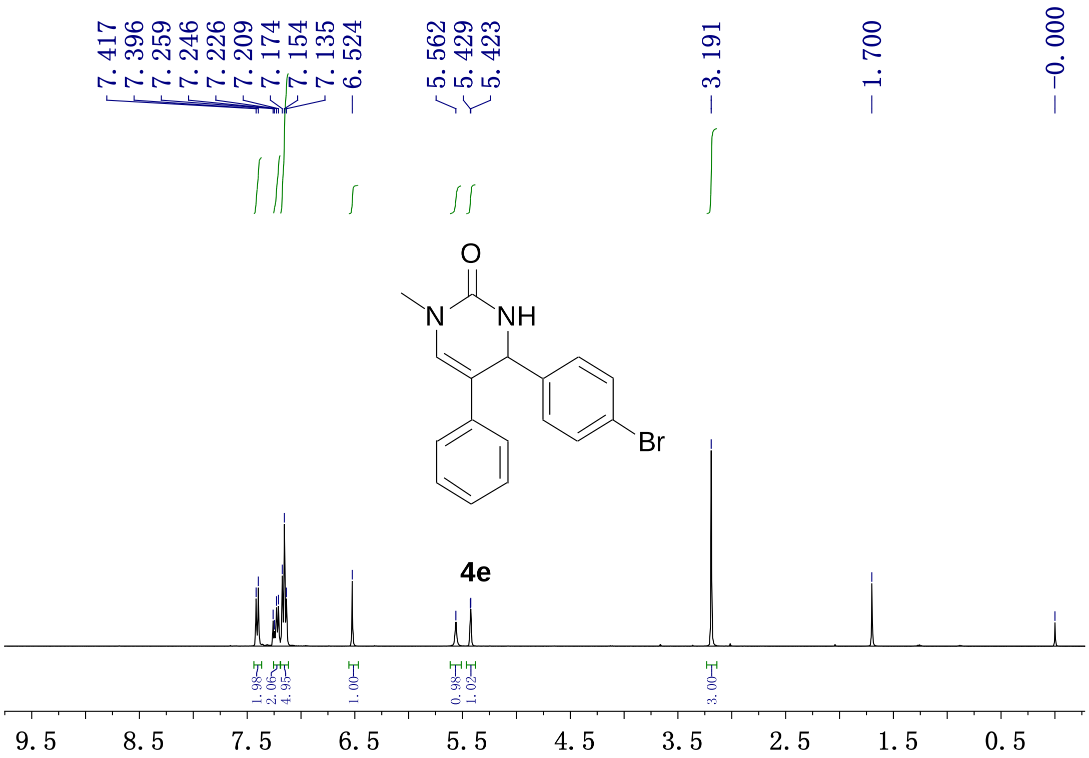
7.417
7.396
7.259
7.246
7.226
7.209
7.174
7.154
7.135
6.524
5.562
5.429
5.423
3.191
1.700
-0.000
O
N
NH
Br
4e
1.98
2.06
4.95
1.00
0.98
1.02
3.00
9.5
8.5
7.5
6.5
5.5
4.5
3.5
2.5
1.5
0.5

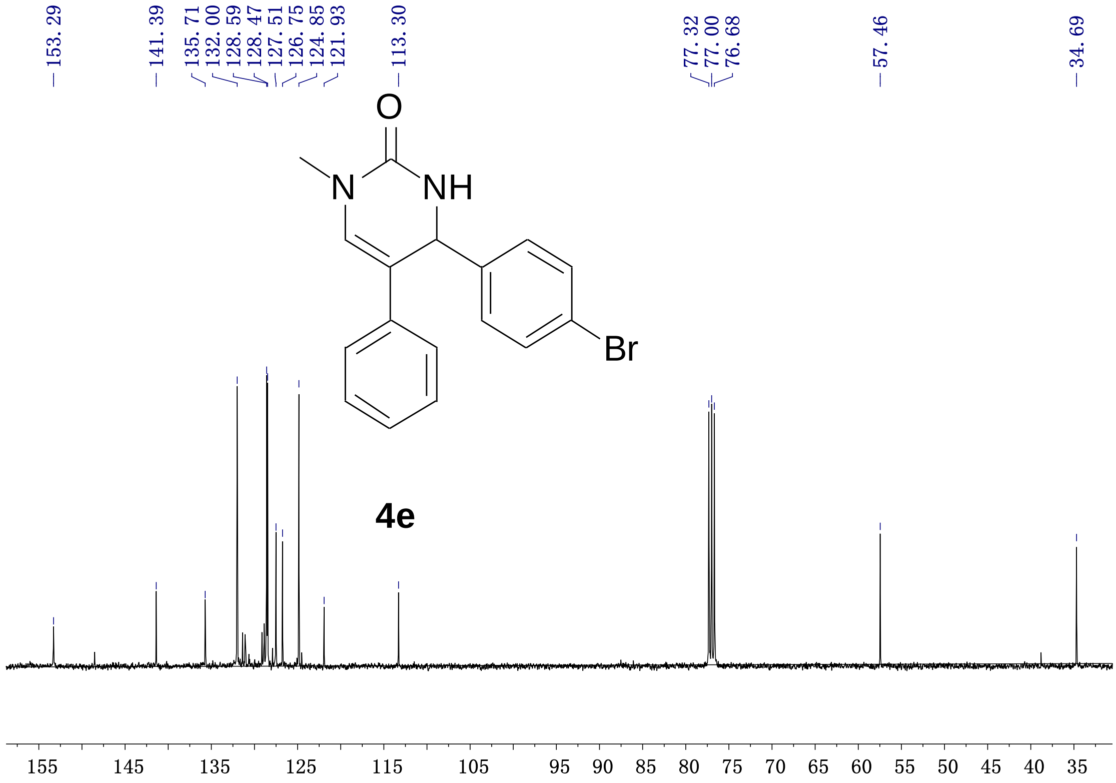
153.29
141.39
135.71
132.00
128.59
128.47
127.51
126.75
124.85
121.93
113.30
77.32
77.00
76.68
57.46
34.69
O
N
NH
Br
4e
155
145
135
125
115
105
95
90
85
80
75
70
65
60
55
50
45
40
35

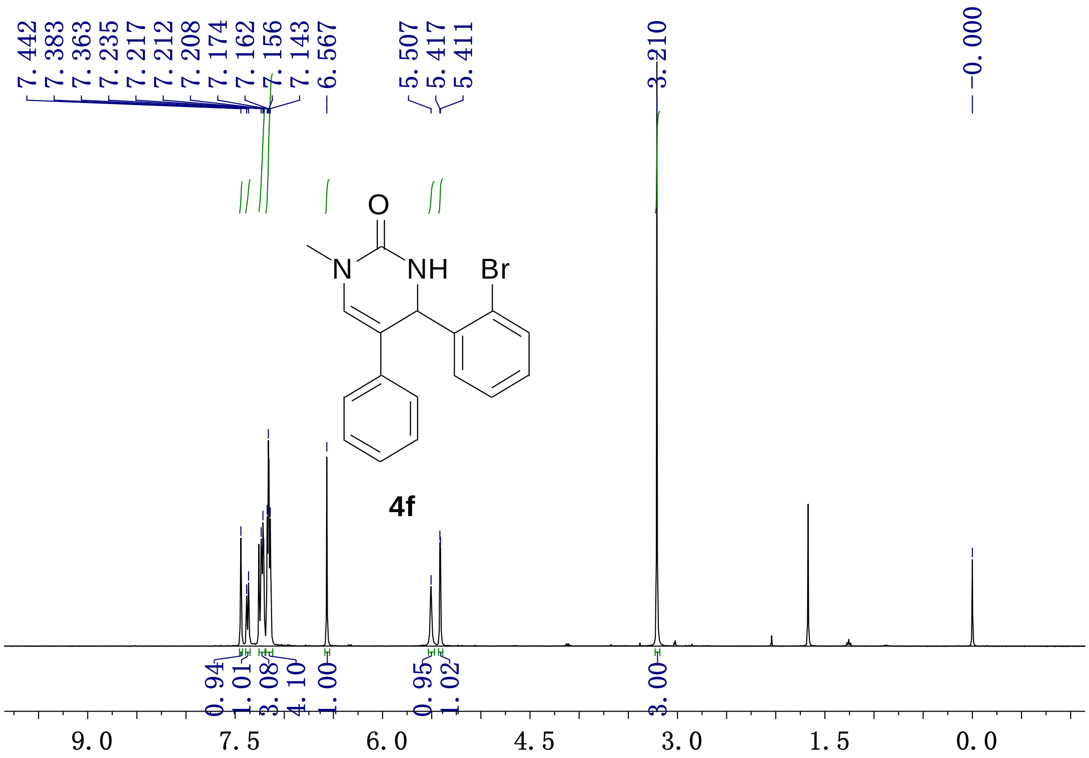
7.442
7.383
7.363
7.235
7.217
7.212
7.208
7.174
7.162
7.156
7.143
6.567
5.507
5.417
5.411
3.210
0.000
O
N
NH
Br
4f
0.94
1.01
3.08
4.10
1.00
0.95
1.02
3.00
9.0
7.5
6.0
4.5
3.0
1.5
0.0

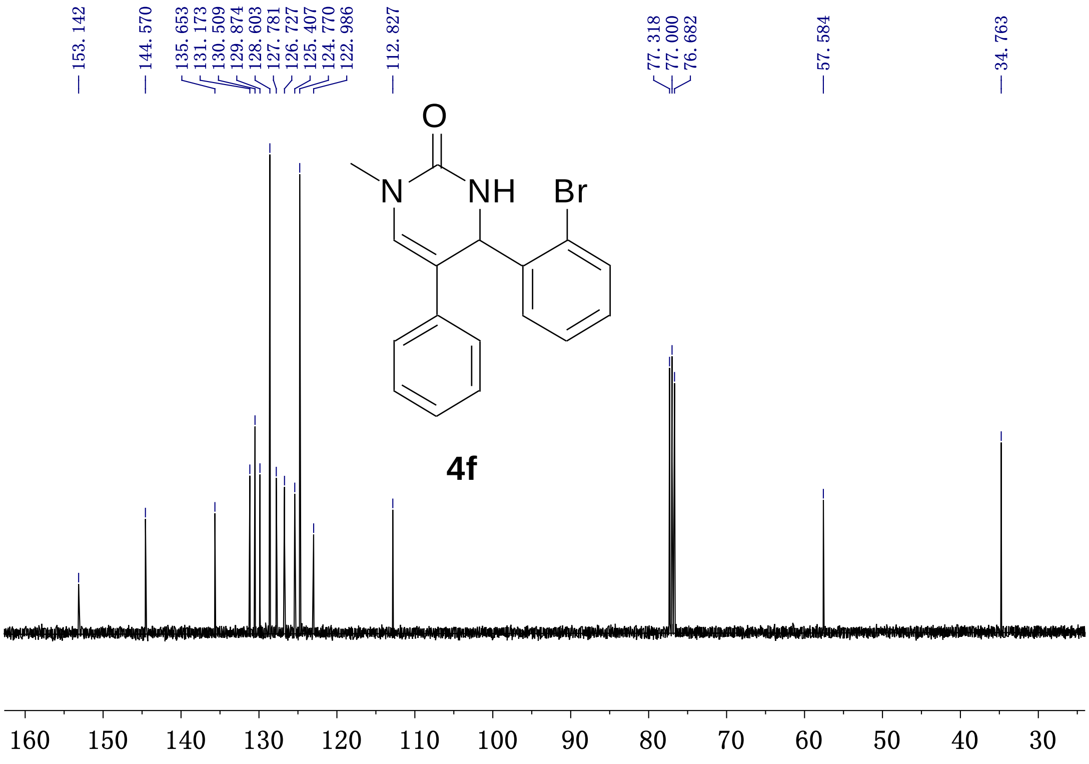
153.142
144.570
135.653
131.173
130.509
129.874
128.603
127.781
126.727
125.407
124.770
122.986
112.827
77.318
77.000
76.682
57.584
34.763
O
N
NH
Br
4f
160
150
140
130
120
110
100
90
80
70
60
50
40
30

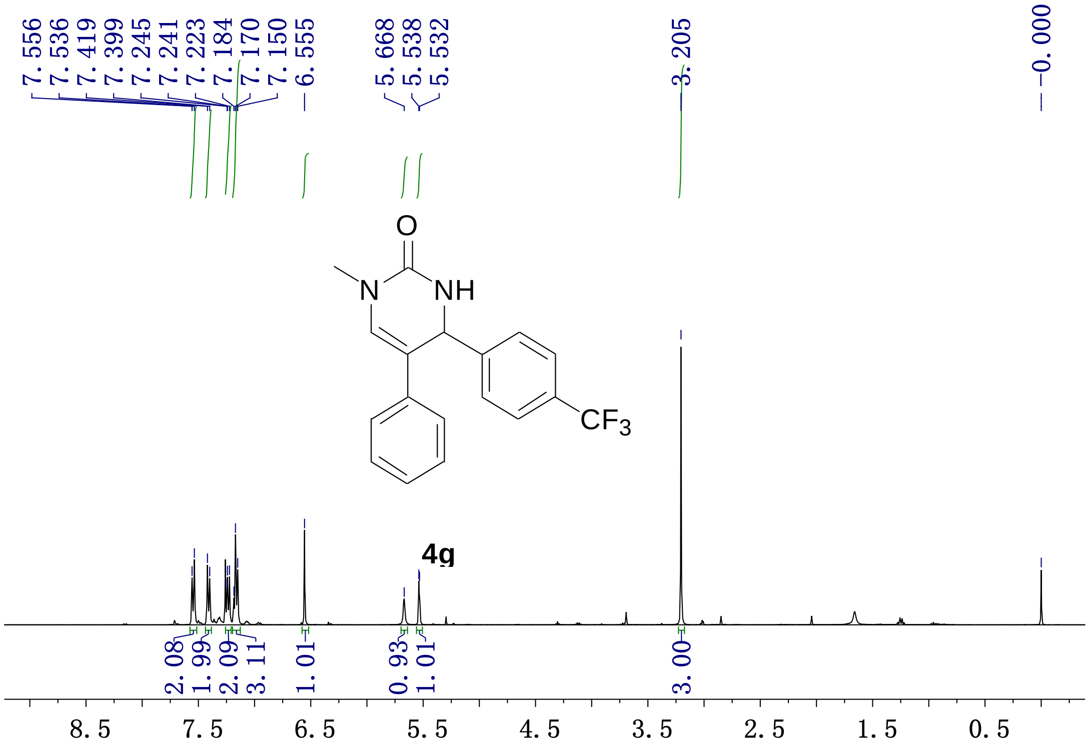
7.556
7.536
7.419
7.399
7.245
7.241
7.223
7.184
7.170
7.150
6.555
5.668
5.538
5.532
3.205
-0.000
O
N
NH
CF3
4g
2.08
1.99
2.09
3.11
1.01
0.93
1.01
3.00
8.5
7.5
6.5
5.5
4.5
3.5
2.5
1.5
0.5

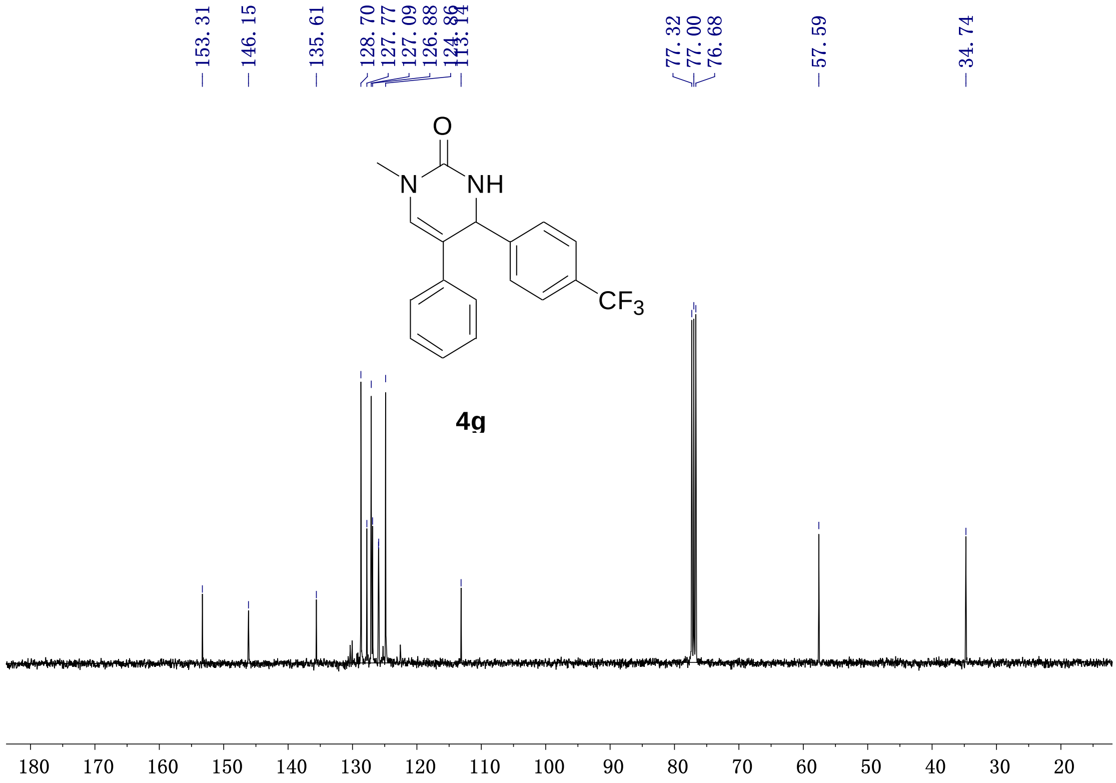
153.31
146.15
135.61
128.70
127.77
127.09
126.88
124.86
113.14
77.32
77.00
76.68
57.59
34.74
O
N
NH
CF3
4g
180
170
160
150
140
130
120
110
100
90
80
70
60
50
40
30
20

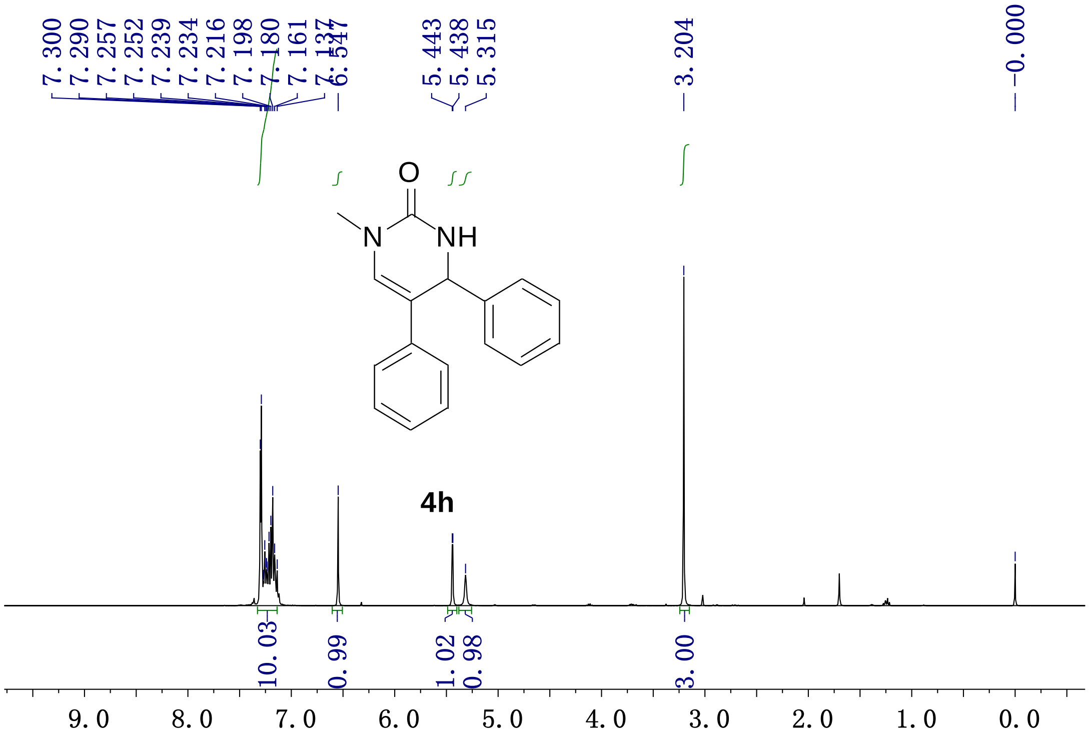
7.300
7.290
7.257
7.252
7.239
7.234
7.216
7.198
7.180
7.161
7.137
6.547
5.443
5.438
5.315
3.204
0.000
O
N
NH
4h
10.03
0.99
1.02
0.98
3.00
9.0
8.0
7.0
6.0
5.0
4.0
3.0
2.0
1.0
0.0

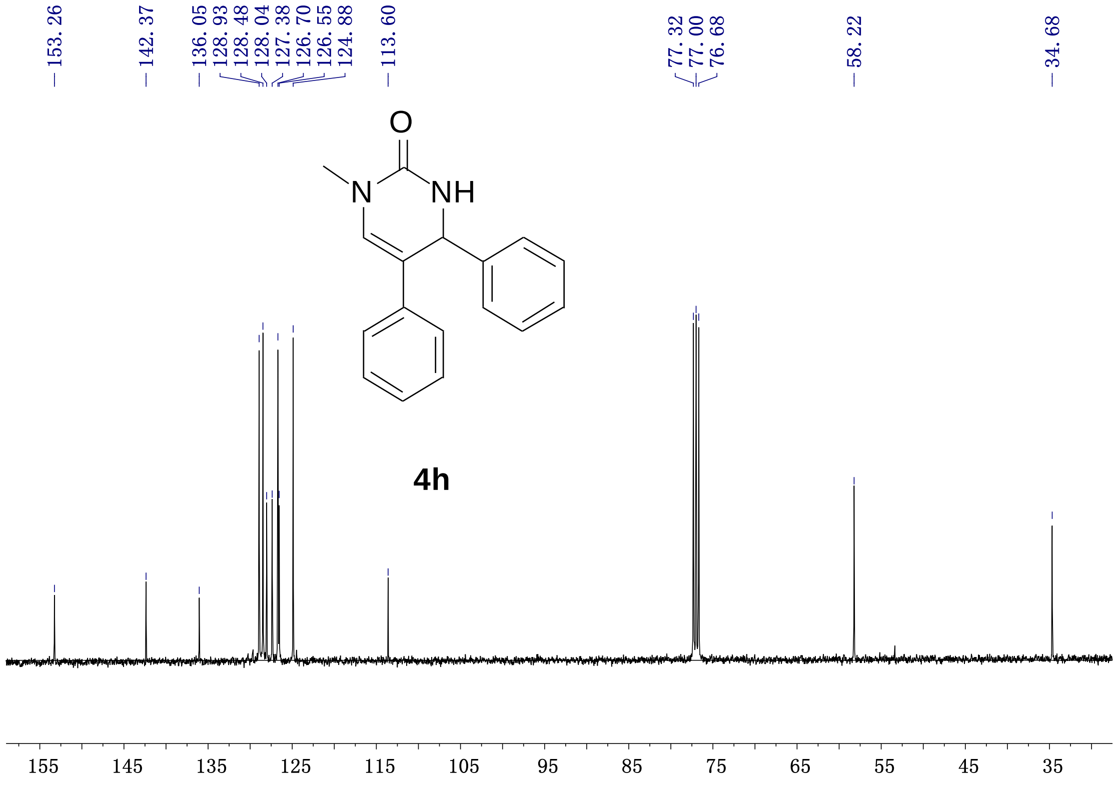
153.26
142.37
136.05
128.93
128.48
128.04
127.38
126.70
126.55
124.88
113.60
77.32
77.00
76.68
58.22
34.68
O
N
NH
4h
155
145
135
125
115
105
95
85
75
65
55
45
35

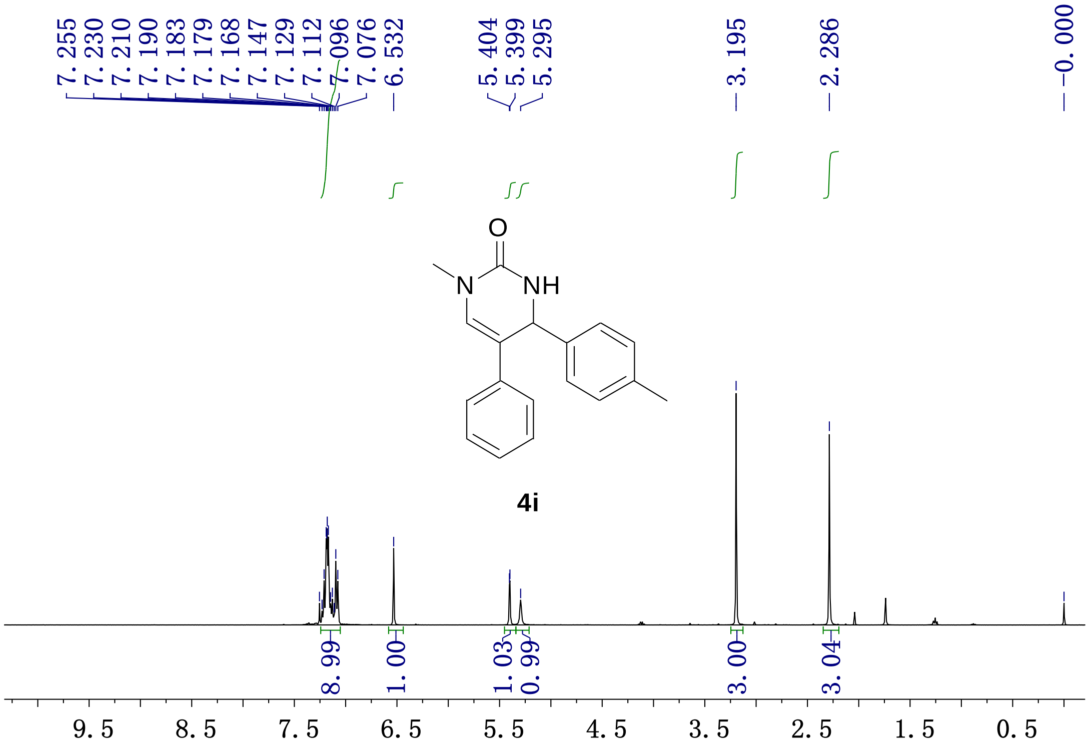

7.255 7.230 7.210 7.190 7.183 7.179 7.168 7.147 7.129 7.112 7.096 7.076 6.532
5.404 5.399 5.295
3.195
2.286
-0.000
O
N
NH
4i
8.99 1.00 1.03 0.99 3.00 3.04
9.5 8.5 7.5 6.5 5.5 4.5 3.5 2.5 1.5 0.5

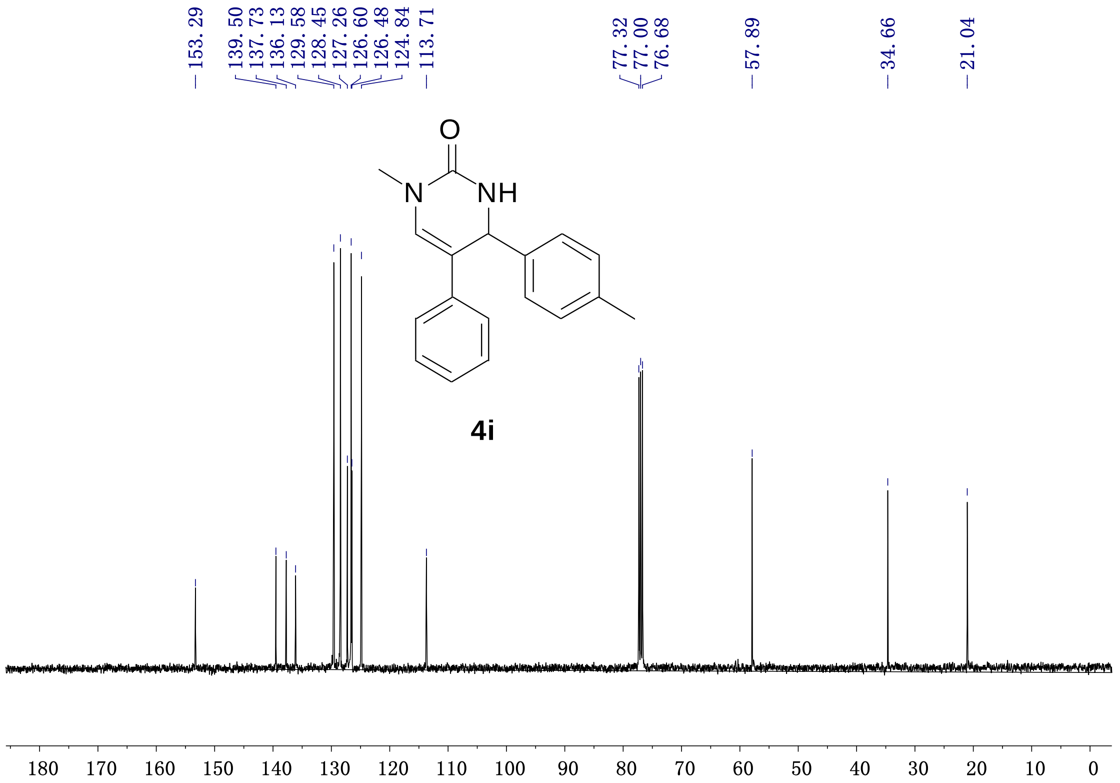

153.29
139.50 137.73 136.13 129.58 128.45 127.26 126.60 126.48 124.84 113.71
77.32 77.00 76.68
57.89
34.66
21.04
O
N
NH
4i
180 170 160 150 140 130 120 110 100 90 80 70 60 50 40 30 20 10 0

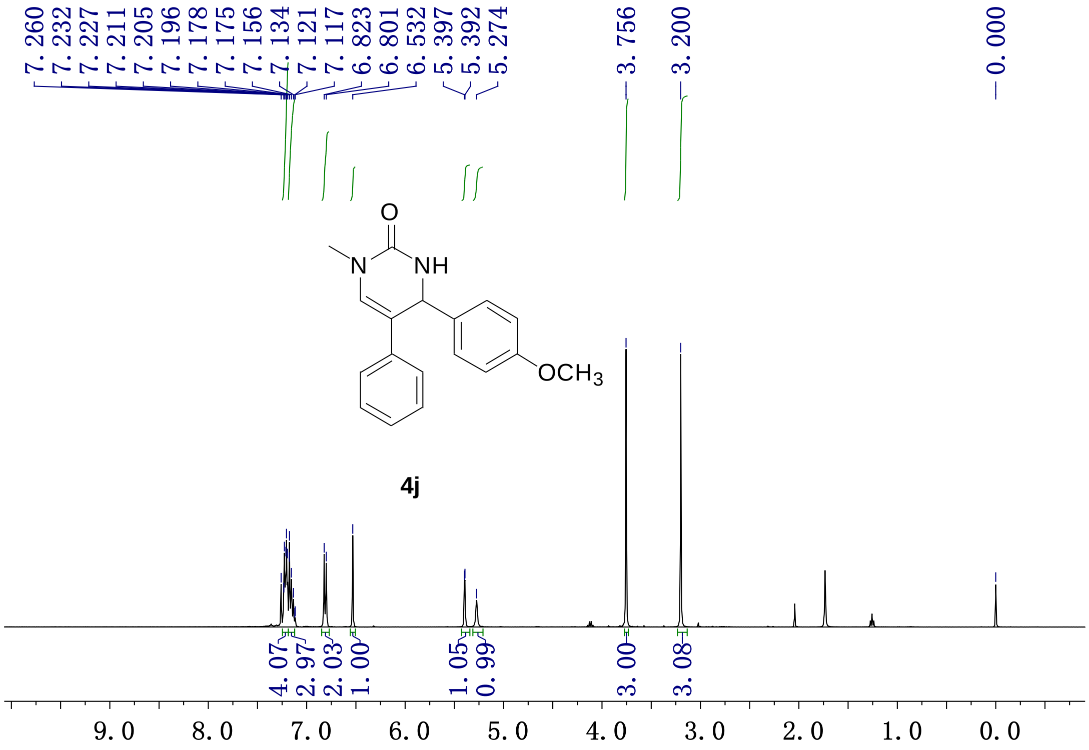
7.260
7.232
7.227
7.211
7.205
7.196
7.178
7.175
7.156
7.134
7.121
7.117
6.823
6.801
6.532
5.397
5.392
5.274
3.756
3.200
0.000
O
N
NH
OCH3
4j
4.07
2.97
2.03
1.00
1.05
0.99
3.00
3.08
9.0
8.0
7.0
6.0
5.0
4.0
3.0
2.0
1.0
0.0

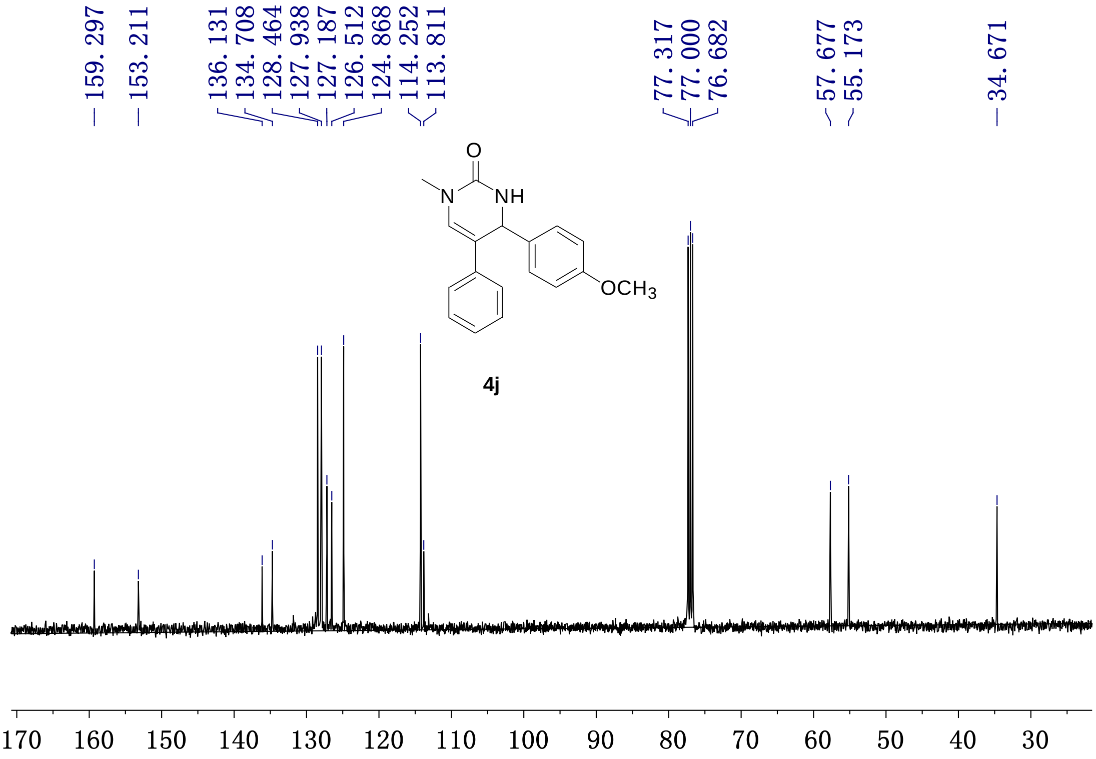
159.297
153.211
136.131
134.708
128.464
127.938
127.187
126.512
124.868
114.252
113.811
77.317
77.000
76.682
57.677
55.173
34.671
O
N
NH
OCH3
4j
170
160
150
140
130
120
110
100
90
80
70
60
50
40
30

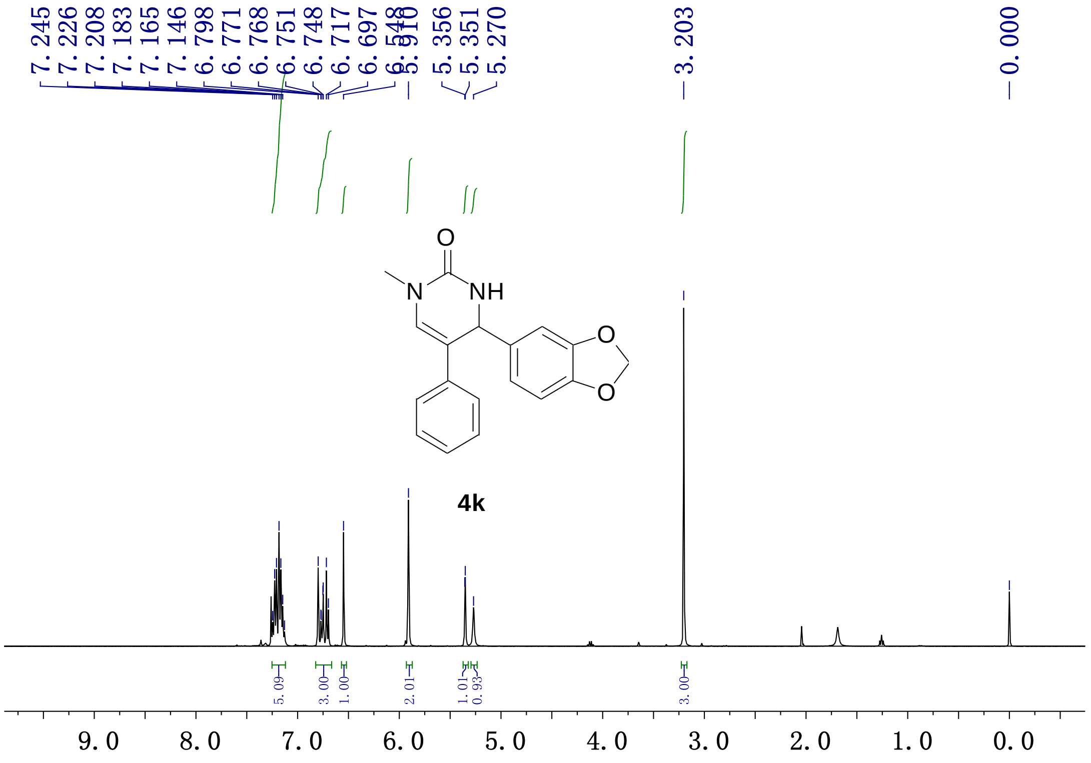
7.245
7.226
7.208
7.183
7.165
7.146
6.798
6.771
6.768
6.751
6.748
6.717
6.697
6.548
5.910
5.356
5.351
5.270
3.203
0.000
O
N
NH
O
O
4k
5.09
3.00
1.00
2.01
1.01
0.93
3.00
9.0
8.0
7.0
6.0
5.0
4.0
3.0
2.0
1.0
0.0

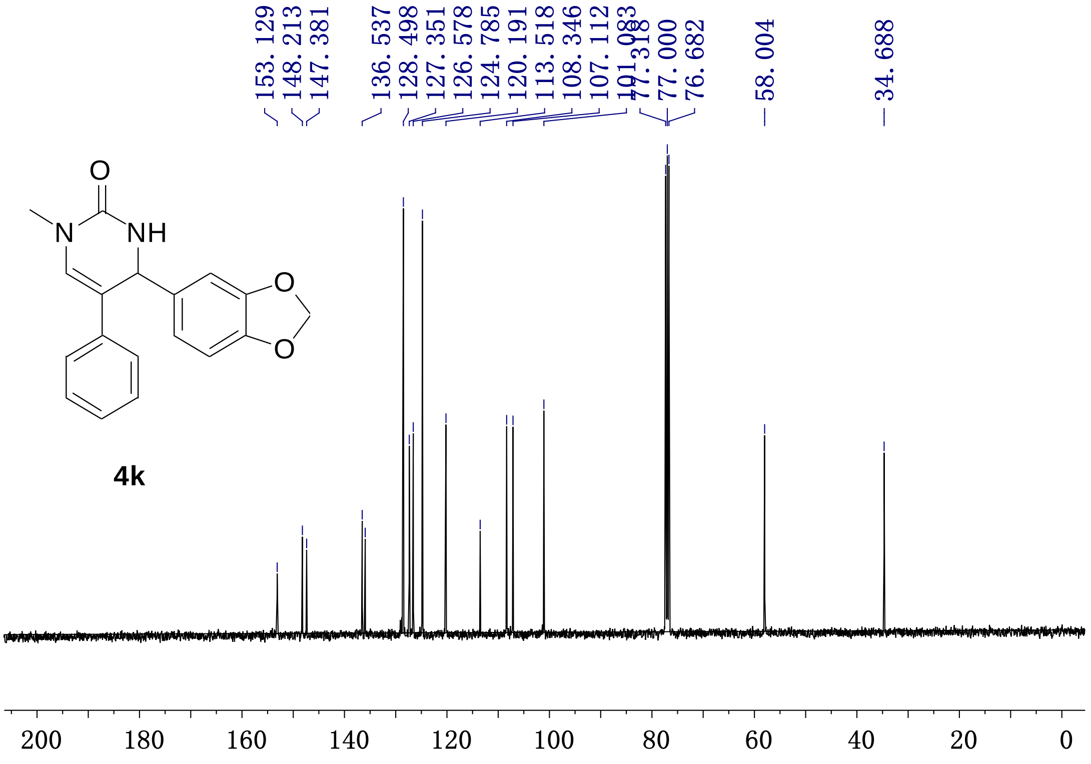
153.129
148.213
147.381
136.537
128.498
127.351
126.578
124.785
120.191
113.518
108.346
107.112
101.083
77.318
77.000
76.682
58.004
34.688
O
N
NH
O
O
4k
200
180
160
140
120
100
80
60
40
20
0

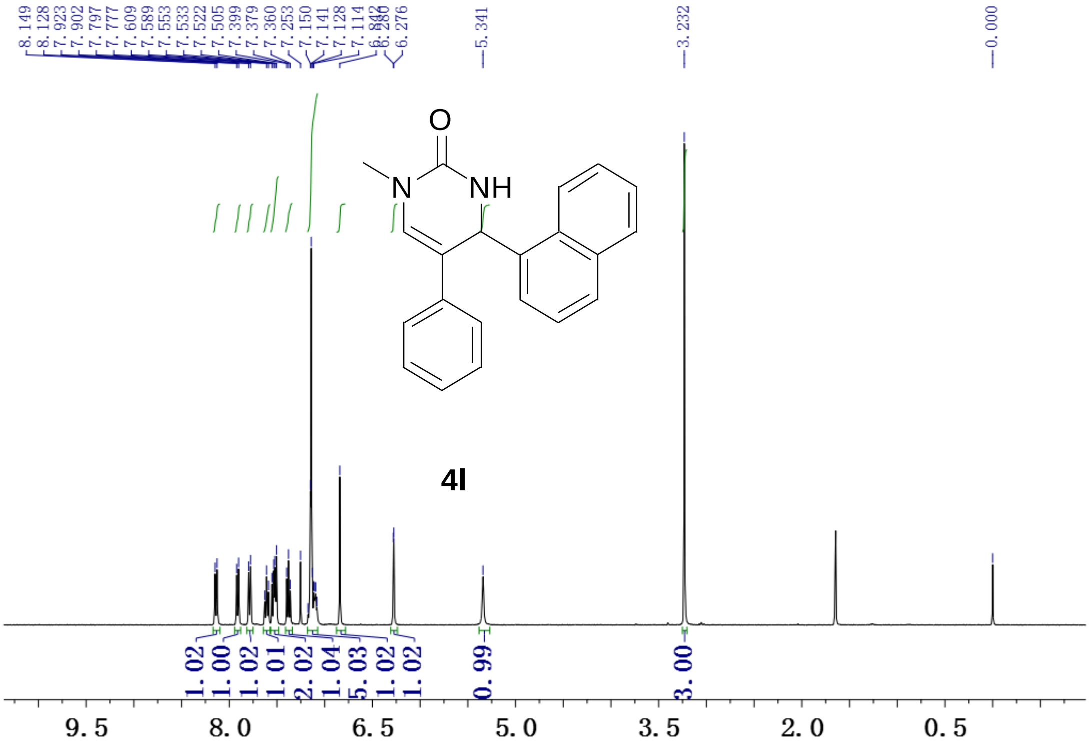
4l

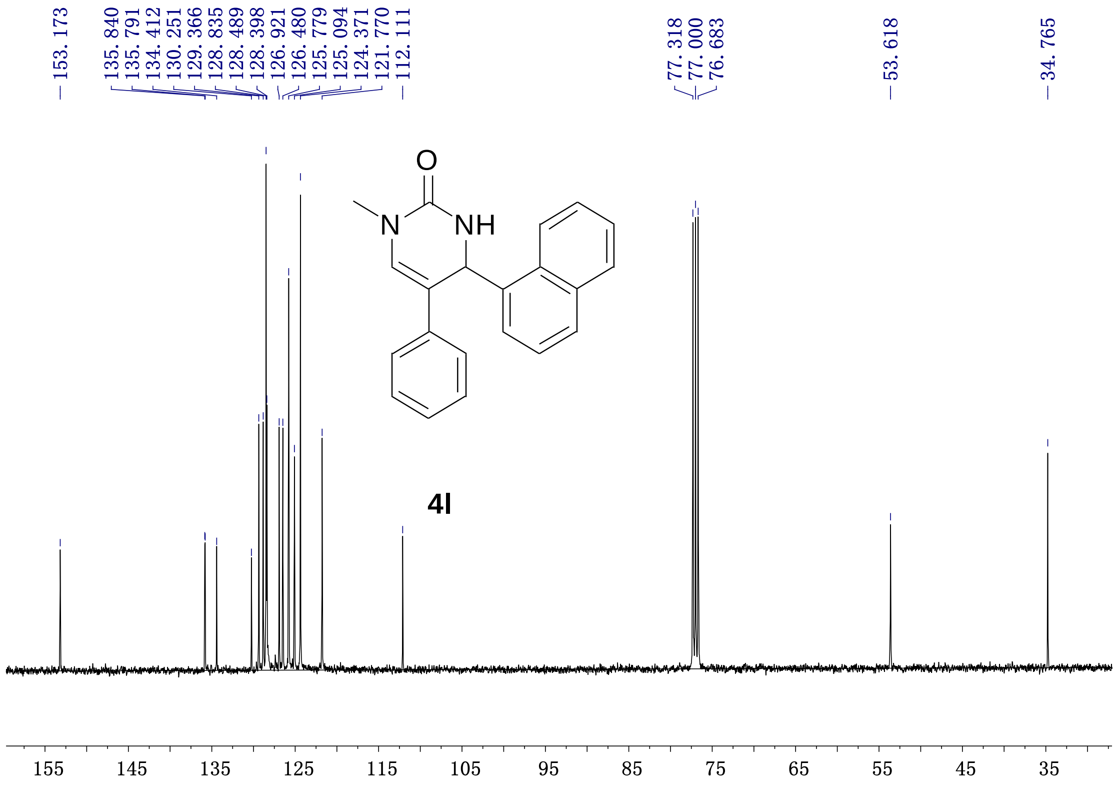
4l

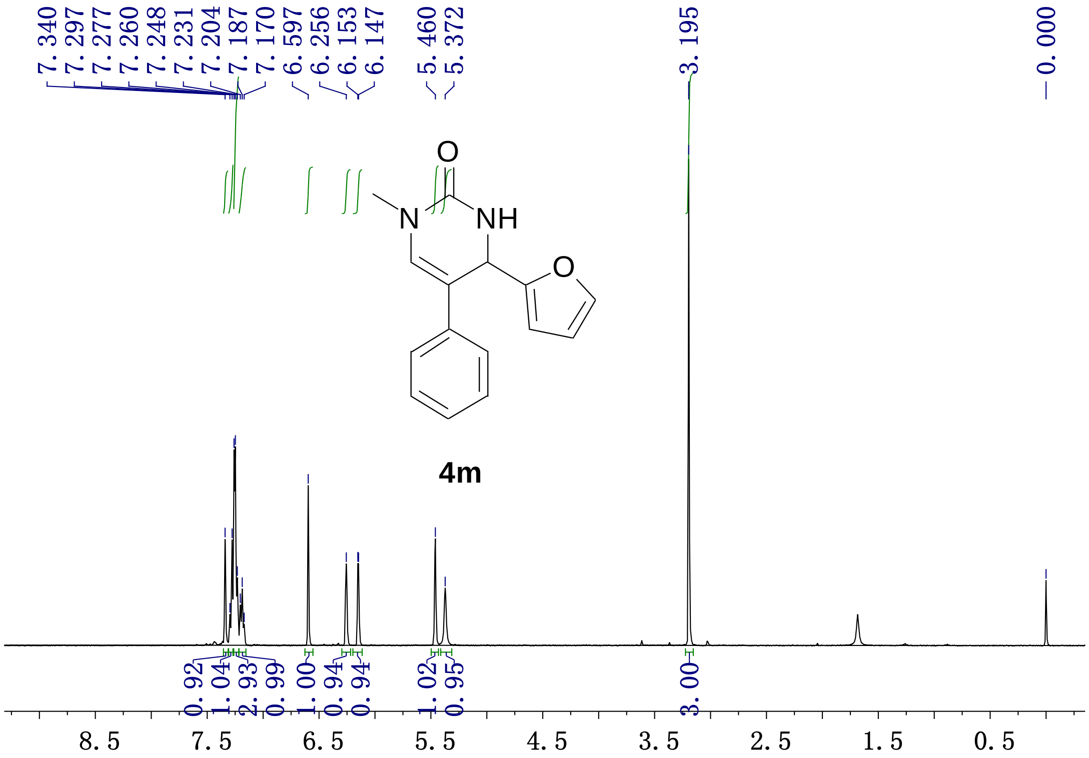
7.340
7.297
7.277
7.260
7.248
7.231
7.204
7.187
7.170
6.597
6.256
6.153
6.147
5.460
5.372
3.195
0.000
O
N
NH
O
4m
0.92
1.04
2.93
0.99
1.00
0.94
0.94
1.02
0.95
3.00
8.5
7.5
6.5
5.5
4.5
3.5
2.5
1.5
0.5

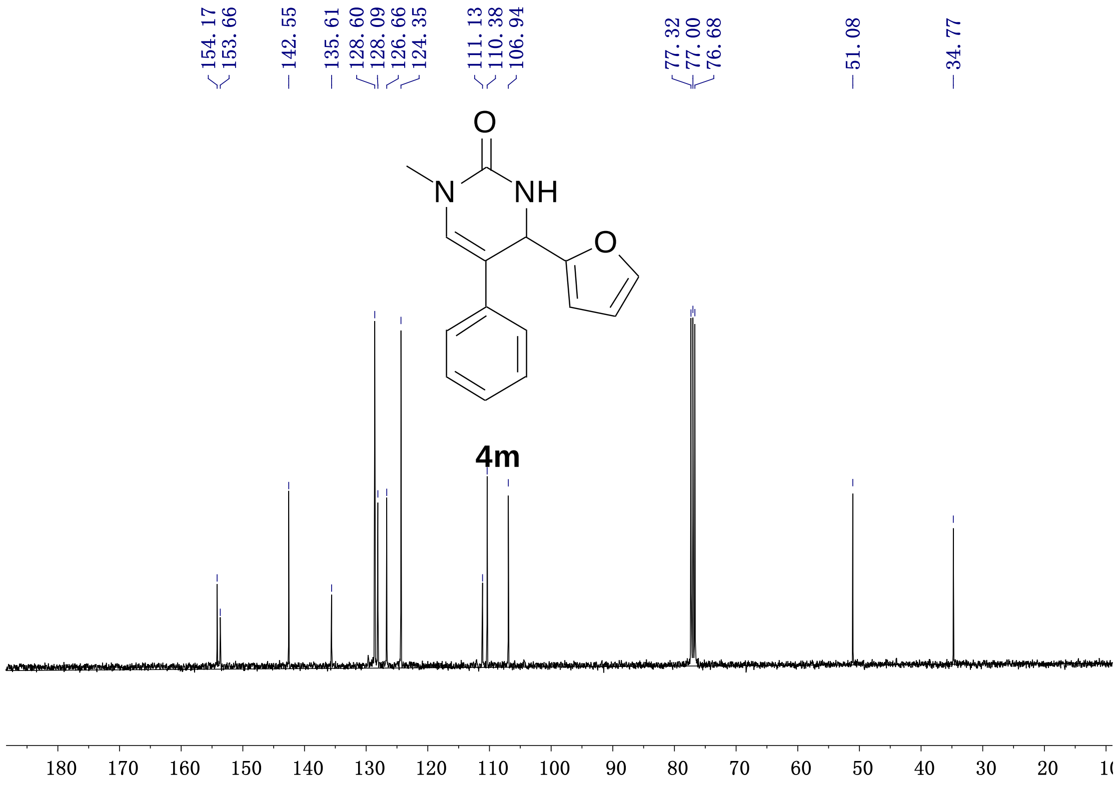
154.17
153.66
142.55
135.61
128.60
128.09
126.66
124.35
111.13
110.38
106.94
77.32
77.00
76.68
51.08
34.77
O
N
NH
O
4m
180
170
160
150
140
130
120
110
100
90
80
70
60
50
40
30
20

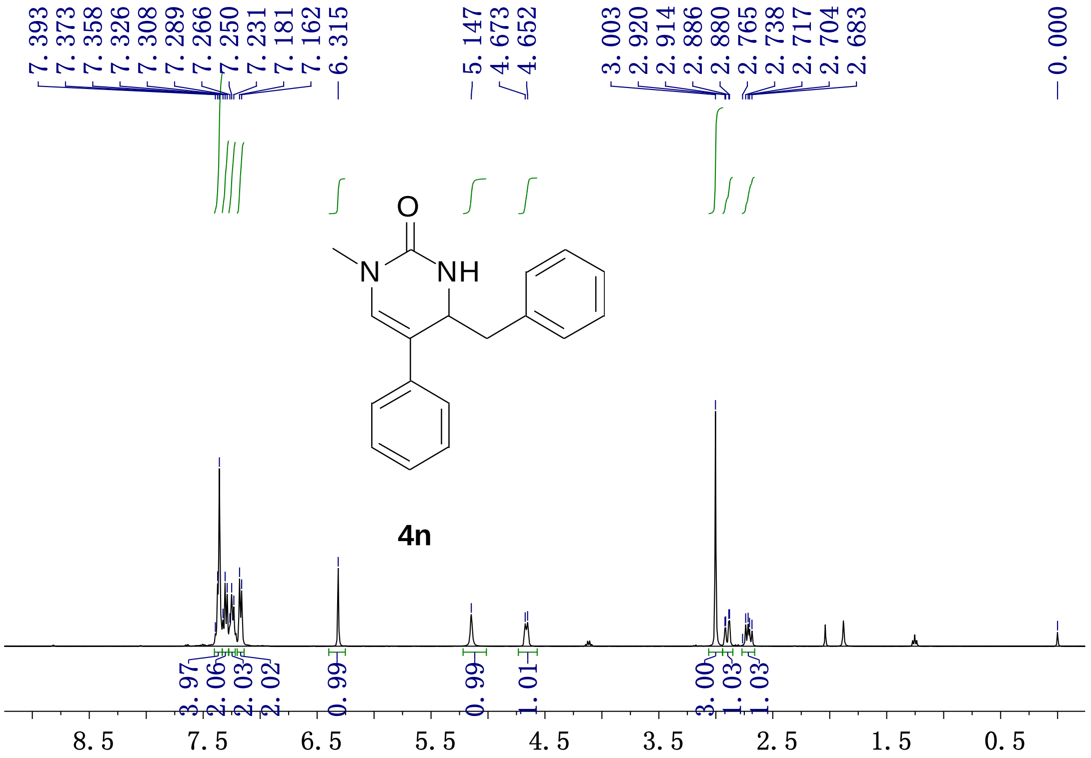
7.393
7.373
7.358
7.326
7.308
7.289
7.266
7.250
7.231
7.181
7.162
6.315
5.147
4.673
4.652
3.003
2.920
2.914
2.886
2.880
2.765
2.738
2.717
2.704
2.683
0.000
O
N
NH
4n
3.97
2.06
2.03
2.02
0.99
0.99
1.01
3.00
1.03
1.03
8.5
7.5
6.5
5.5
4.5
3.5
2.5
1.5
0.5

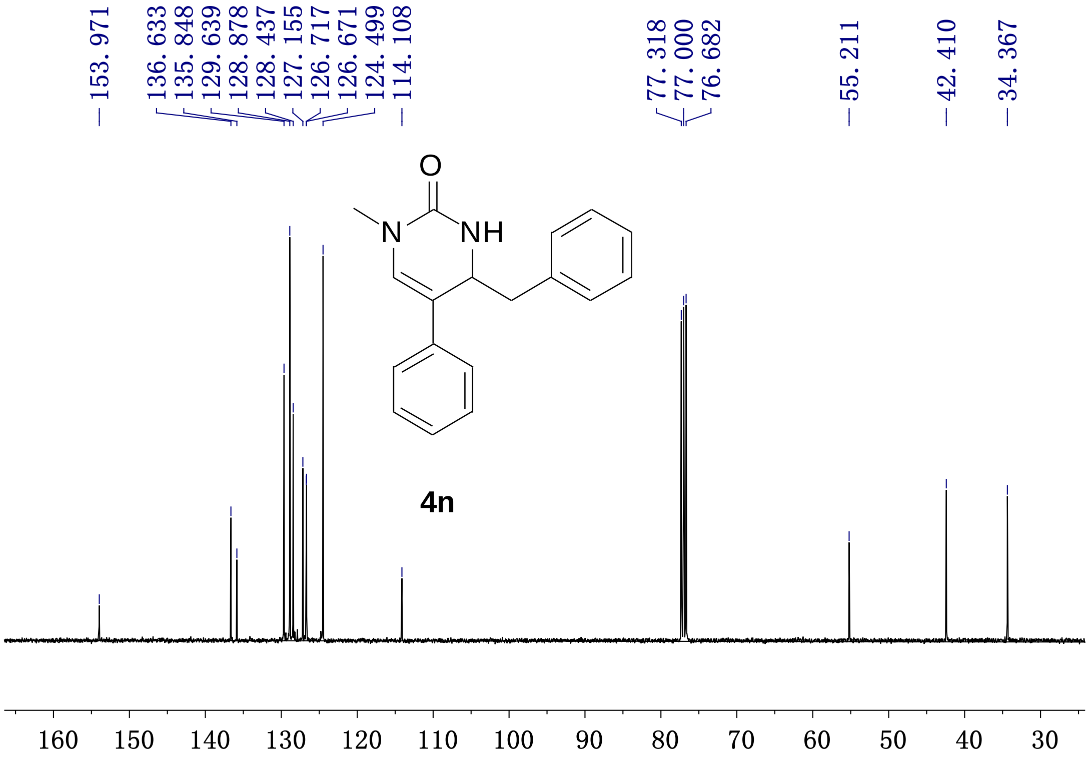
153.971
136.633
135.848
129.639
128.878
128.437
127.155
126.717
126.671
124.499
114.108
77.318
77.000
76.682
55.211
42.410
34.367
O
N
NH
4n
160
150
140
130
120
110
100
90
80
70
60
50
40
30

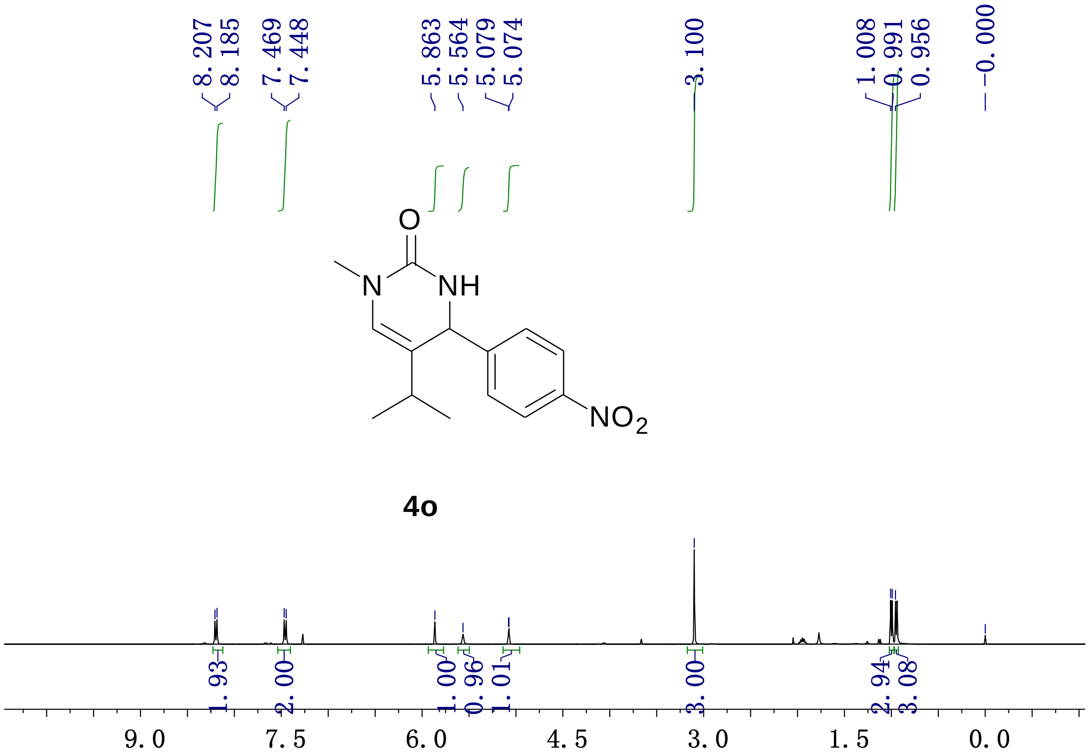
8.207
8.185
7.469
7.448
5.863
5.564
5.079
5.074
3.100
1.008
0.991
0.956
-0.000
O
N
NH
NO2
4o
1.93
2.00
1.00
0.96
1.01
3.00
2.94
3.08
9.0
7.5
6.0
4.5
3.0
1.5
0.0

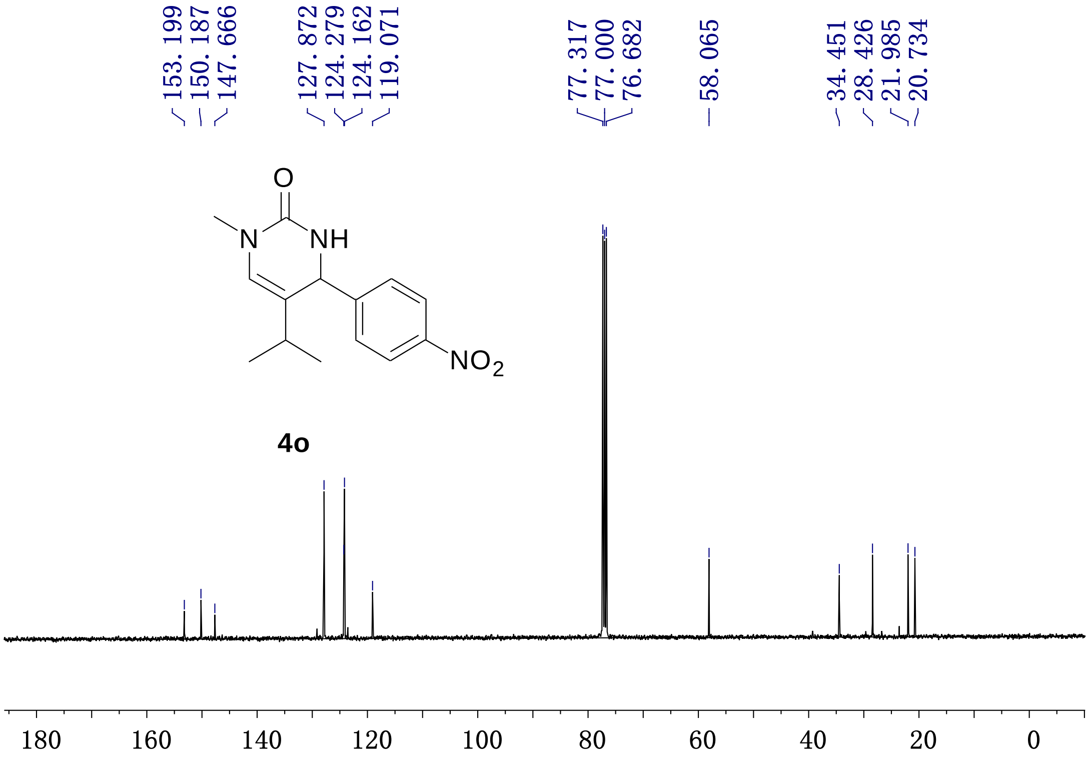
153.199
150.187
147.666
127.872
124.279
124.162
119.071
77.317
77.000
76.682
58.065
34.451
28.426
21.985
20.734
O
N
NH
NO2
4o
180
160
140
120
100
80
60
40
20
0

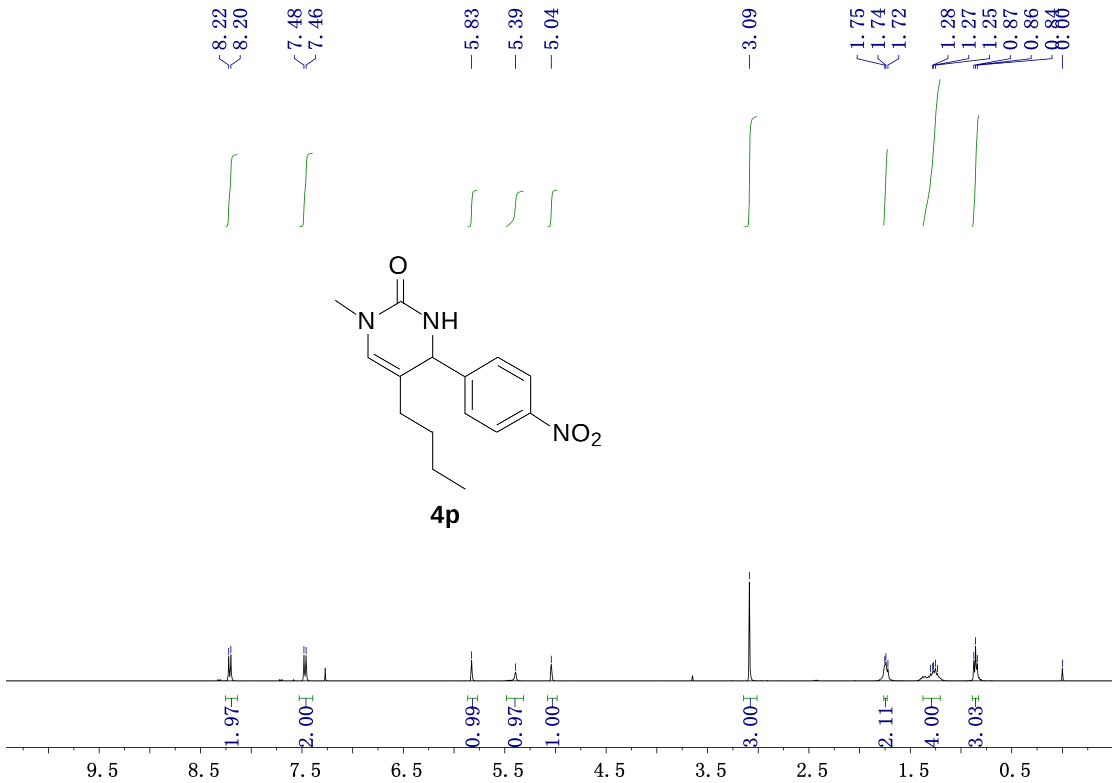
8.22
8.20
7.48
7.46
5.83
5.39
5.04
3.09
1.75
1.74
1.72
1.28
1.27
1.25
0.87
0.86
0.84
0.00
O
N
NH
NO2
4p
1.97
2.00
0.99
0.97
1.00
3.00
2.11
4.00
3.03
9.5
8.5
7.5
6.5
5.5
4.5
3.5
2.5
1.5
0.5

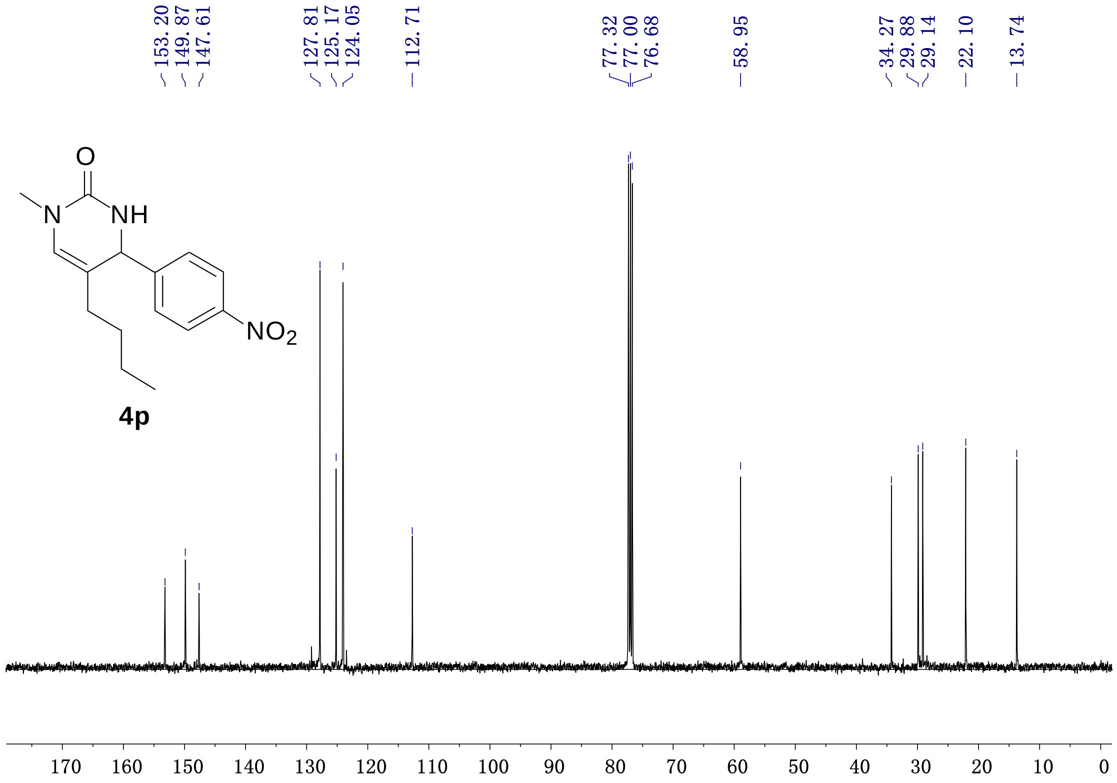
153.20
149.87
147.61
127.81
125.17
124.05
112.71
77.32
77.00
76.68
58.95
34.27
29.88
29.14
22.10
13.74
O
N
NH
NO2
4p
170
160
150
140
130
120
110
100
90
80
70
60
50
40
30
20
10
0

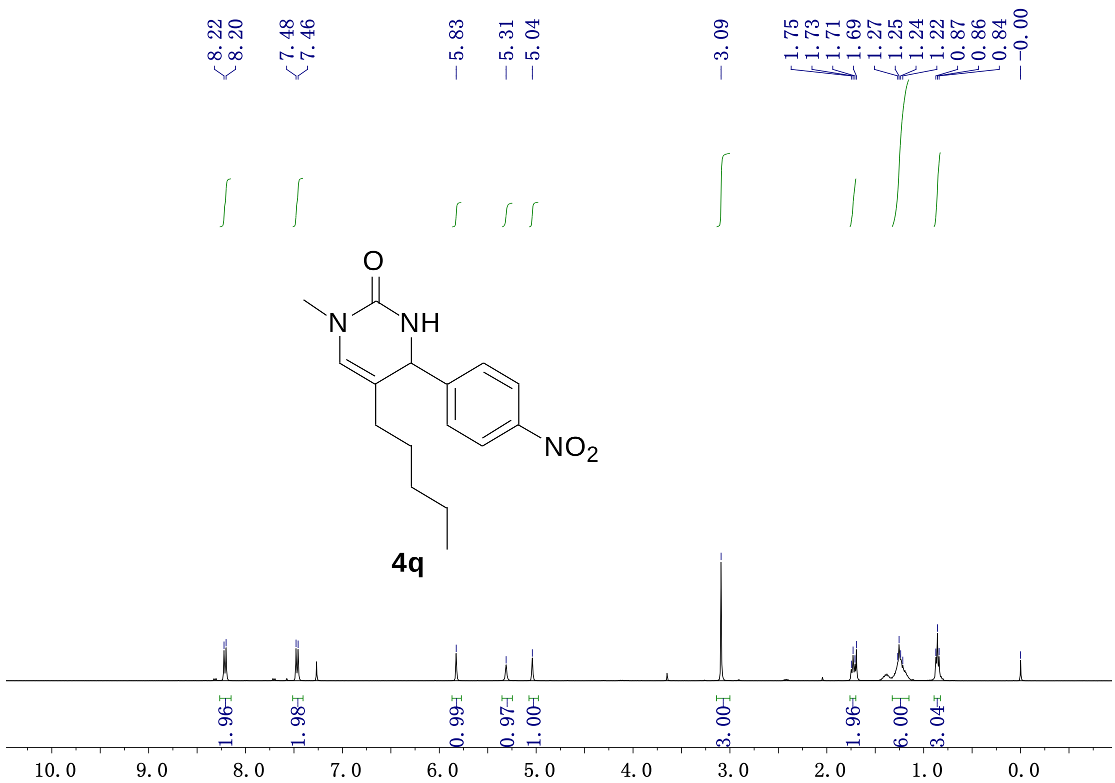

8.22
8.20
7.48
7.46
5.83
5.31
5.04
3.09
1.75
1.73
1.71
1.69
1.27
1.25
1.24
1.22
0.87
0.86
0.84
-0.00
O
N
NH
NO2
4q
1.96
1.98
0.99
0.97
1.00
3.00
1.96
6.00
3.04
10.0
9.0
8.0
7.0
6.0
5.0
4.0
3.0
2.0
1.0
0.0

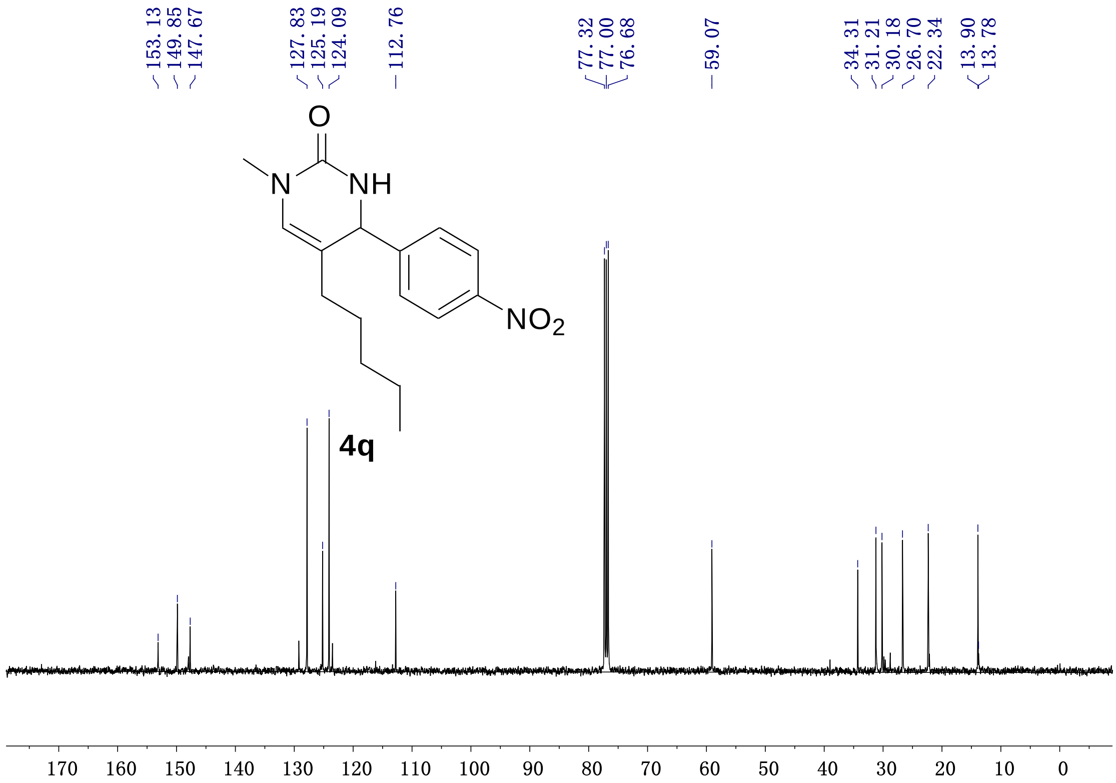

153.13
149.85
147.67
127.83
125.19
124.09
112.76
77.32
77.00
76.68
59.07
34.31
31.21
30.18
26.70
22.34
13.90
13.78
O
N
NH
NO2
4q
170
160
150
140
130
120
110
100
90
80
70
60
50
40
30
20
10
0

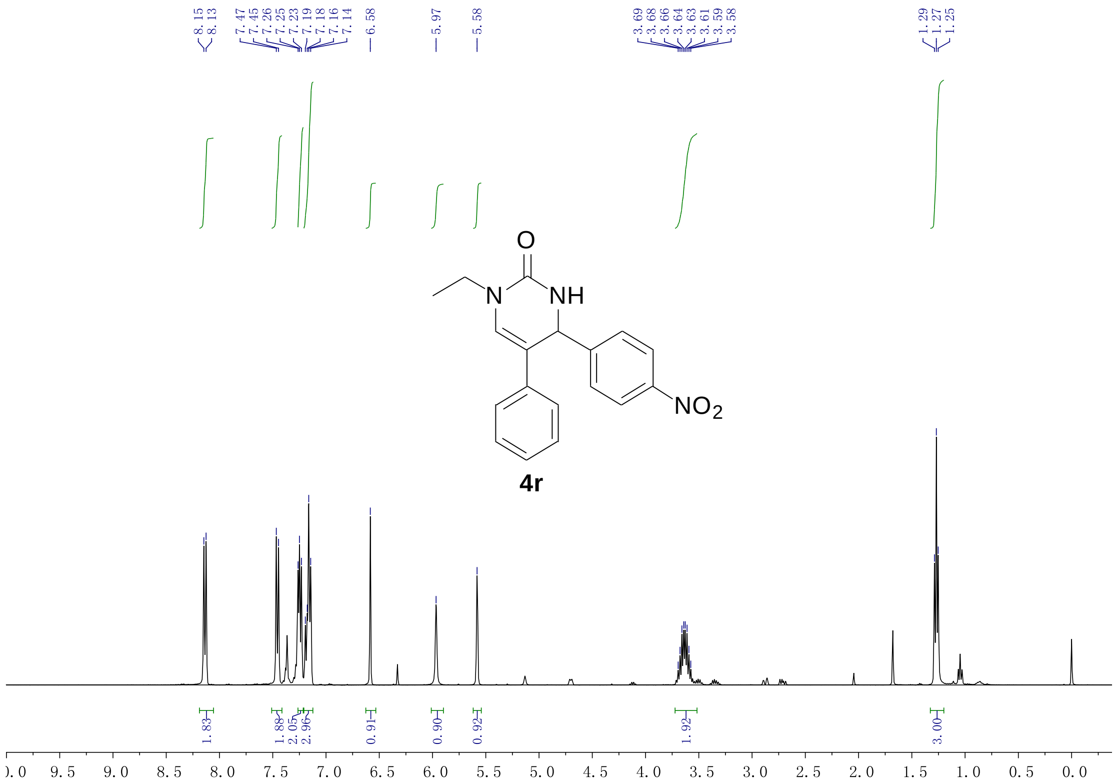
8.15
8.13
7.47
7.45
7.26
7.25
7.23
7.19
7.18
7.16
7.14
6.58
5.97
5.58
3.69
3.68
3.66
3.64
3.63
3.61
3.59
3.58
1.29
1.27
1.25
O
N
NH
NO2
4r
1.83
1.88
2.05
2.96
0.91
0.90
0.92
1.92
3.00
9.5 9.0 8.5 8.0 7.5 7.0 6.5 6.0 5.5 5.0 4.5 4.0 3.5 3.0 2.5 2.0 1.5 1.0 0.5 0.0

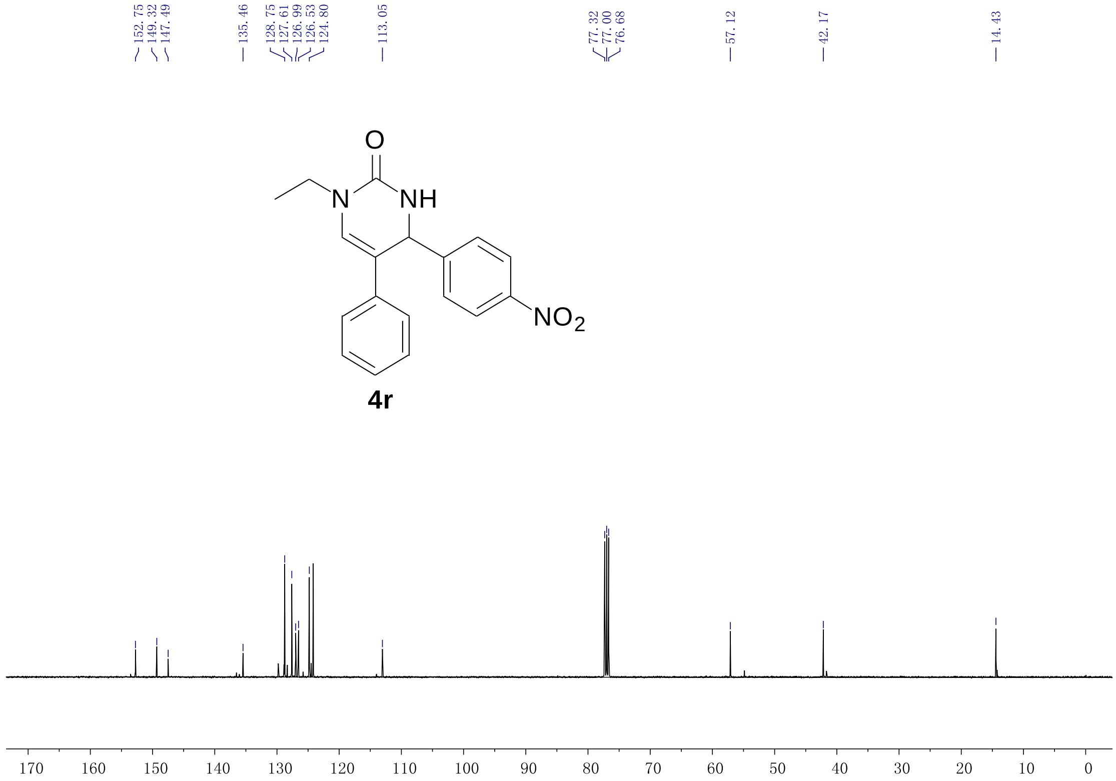
152.75
149.32
147.49
135.46
128.75
127.61
126.99
126.53
124.80
113.05
77.32
77.00
76.68
57.12
42.17
14.43
O
N
NH
NO2
4r
170 160 150 140 130 120 110 100 90 80 70 60 50 40 30 20 10 0

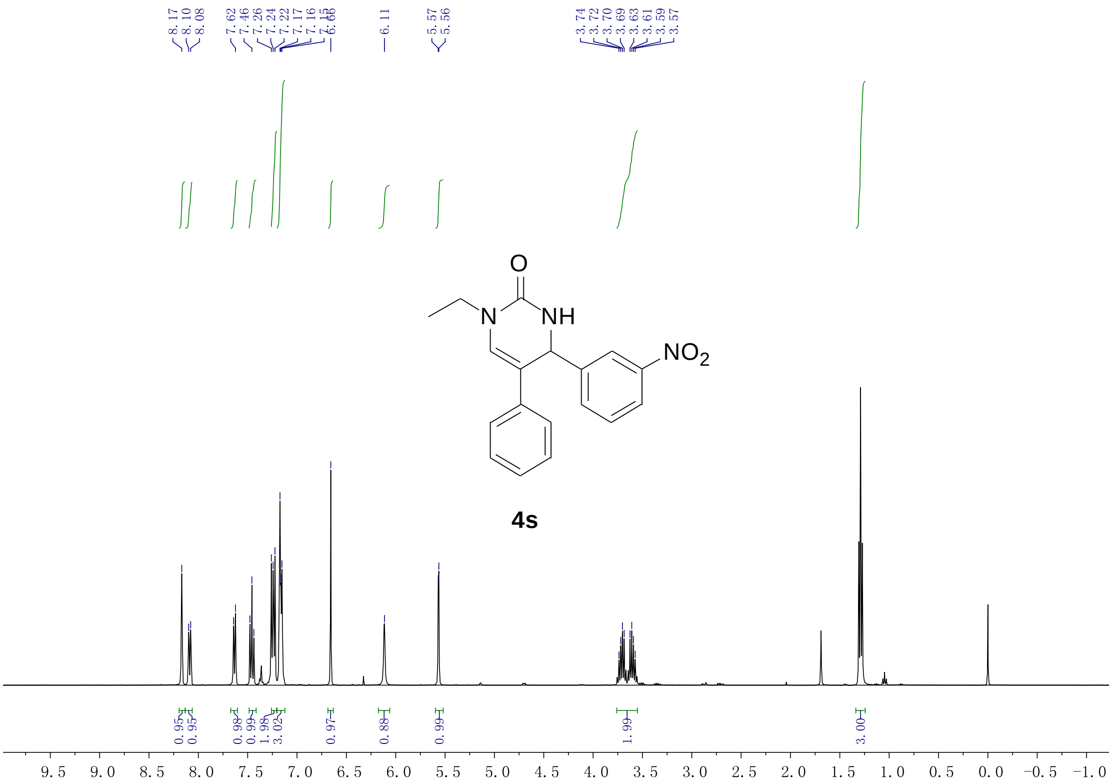
8.17
8.10
8.08
7.62
7.46
7.26
7.24
7.22
7.17
7.16
7.15
6.66
6.11
5.57
5.56
3.74
3.72
3.70
3.69
3.63
3.61
3.59
3.57
O
N
NH
NO2
4s
0.95
0.95
0.98
0.99
1.98
3.02
0.97
0.88
0.99
1.99
3.00
9.5 9.0 8.5 8.0 7.5 7.0 6.5 6.0 5.5 5.0 4.5 4.0 3.5 3.0 2.5 2.0 1.5 1.0 0.5 0.0 -0.5 -1.0

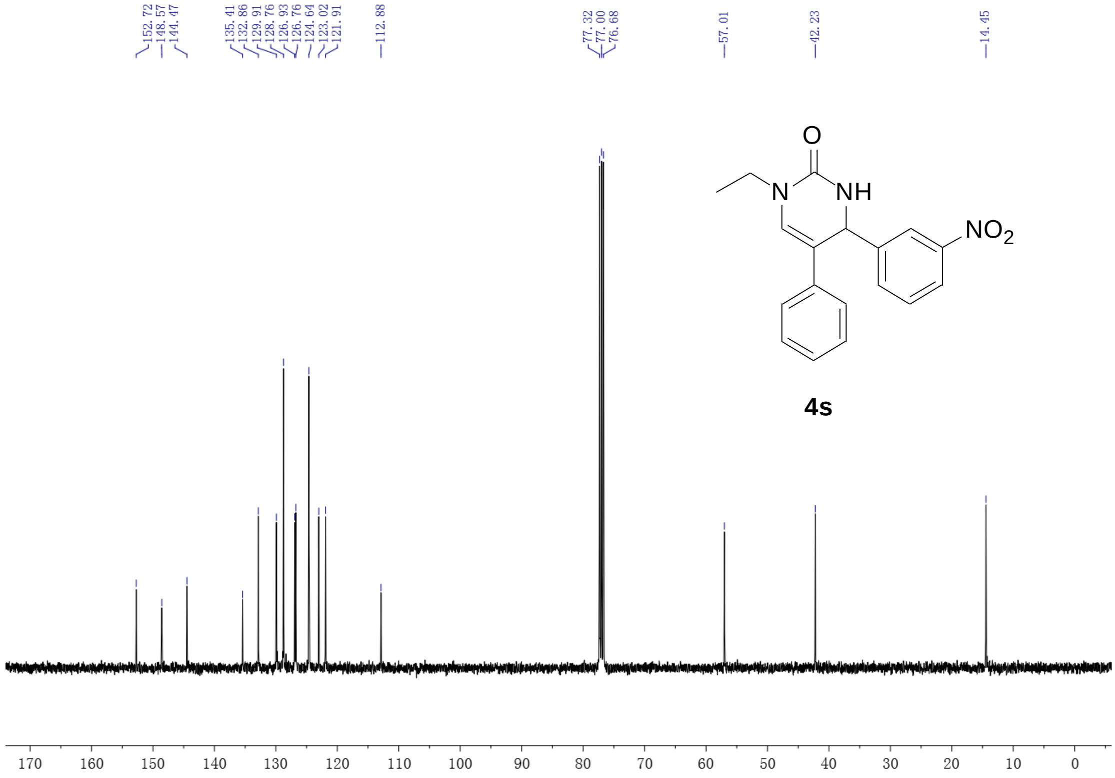
152.72
148.57
144.47
135.41
132.86
129.91
128.76
126.93
126.76
124.64
123.02
121.91
112.88
77.32
77.00
76.68
57.01
42.23
14.45
O
N
NH
NO2
4s
170 160 150 140 130 120 110 100 90 80 70 60 50 40 30 20 10 0

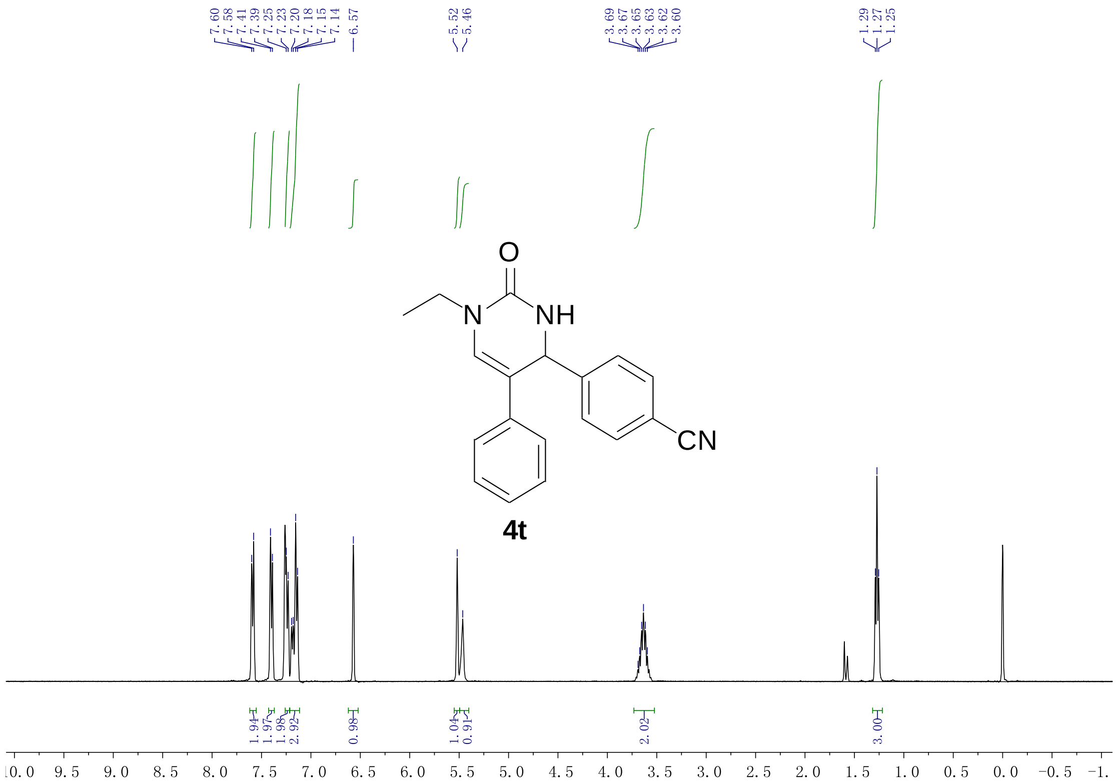
7.60 7.58 7.41 7.39 7.25 7.23 7.20 7.18 7.15 7.14 6.57
5.52 5.46
3.69 3.67 3.65 3.63 3.62 3.60
1.29 1.27 1.25
O
N
NH
CN
4t
1.94 1.97 1.98 2.92 0.98 1.04 0.91 2.02 3.00
10.0 9.5 9.0 8.5 8.0 7.5 7.0 6.5 6.0 5.5 5.0 4.5 4.0 3.5 3.0 2.5 2.0 1.5 1.0 0.5 0.0 −0.5 −1.

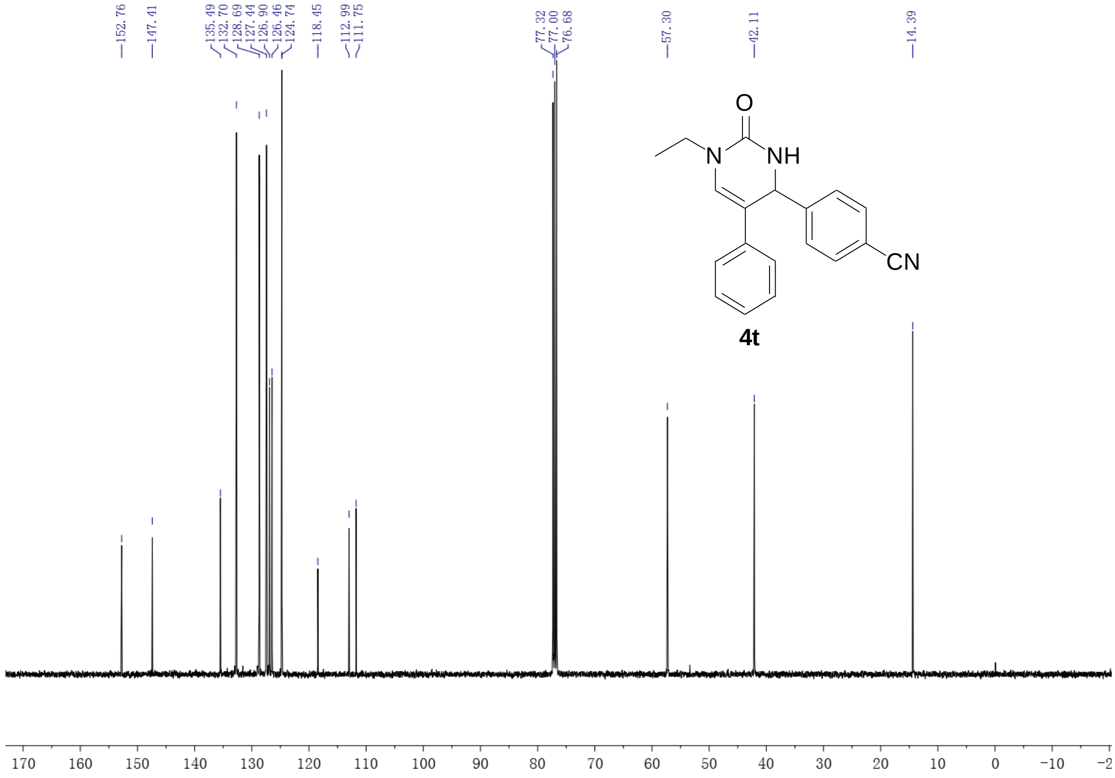
152.76 147.41 135.49 132.70 128.69 127.44 126.90 126.46 124.74 118.45 112.99 111.75
77.32 77.00 76.68 57.30 42.11 14.39
O
N
NH
CN
4t
170 160 150 140 130 120 110 100 90 80 70 60 50 40 30 20 10 0 −10 −2

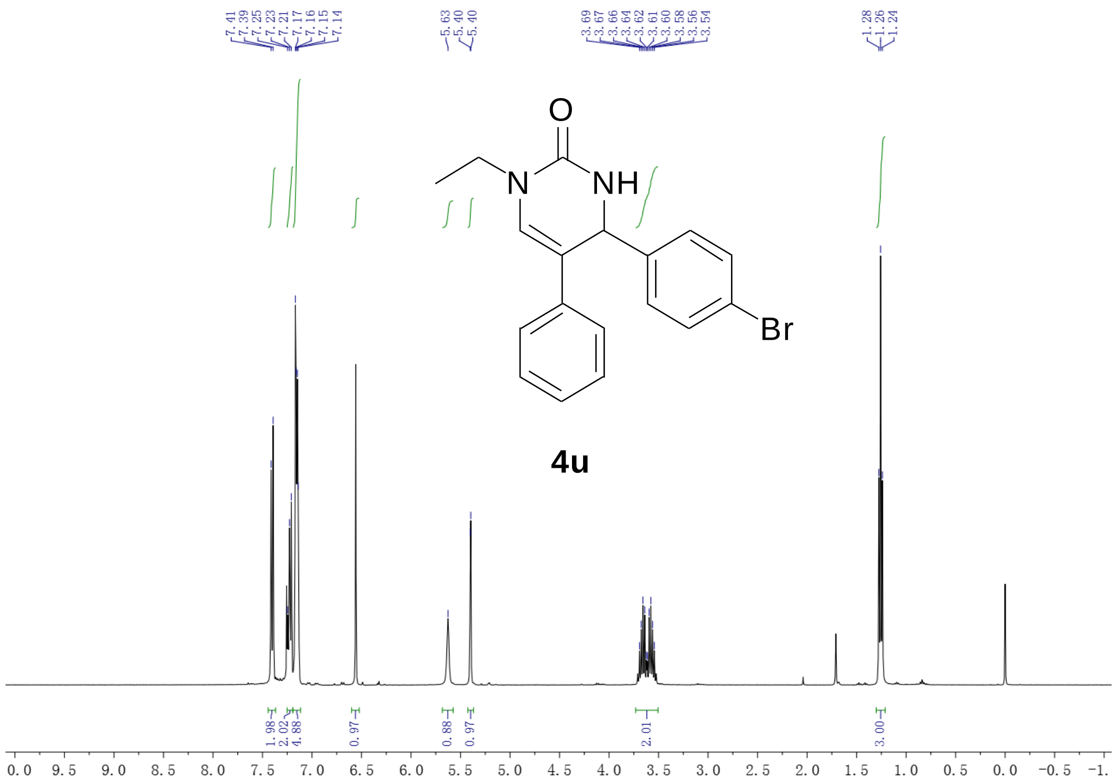
7.41 7.39 7.25 7.23 7.21 7.17 7.16 7.15 7.14
5.63 5.40 5.40
3.69 3.67 3.66 3.64 3.62 3.61 3.60 3.58 3.56 3.54
1.28 1.26 1.24
O
N
NH
Br
4u
1.98 2.02 4.88 0.97 0.88 0.97 2.01 3.00

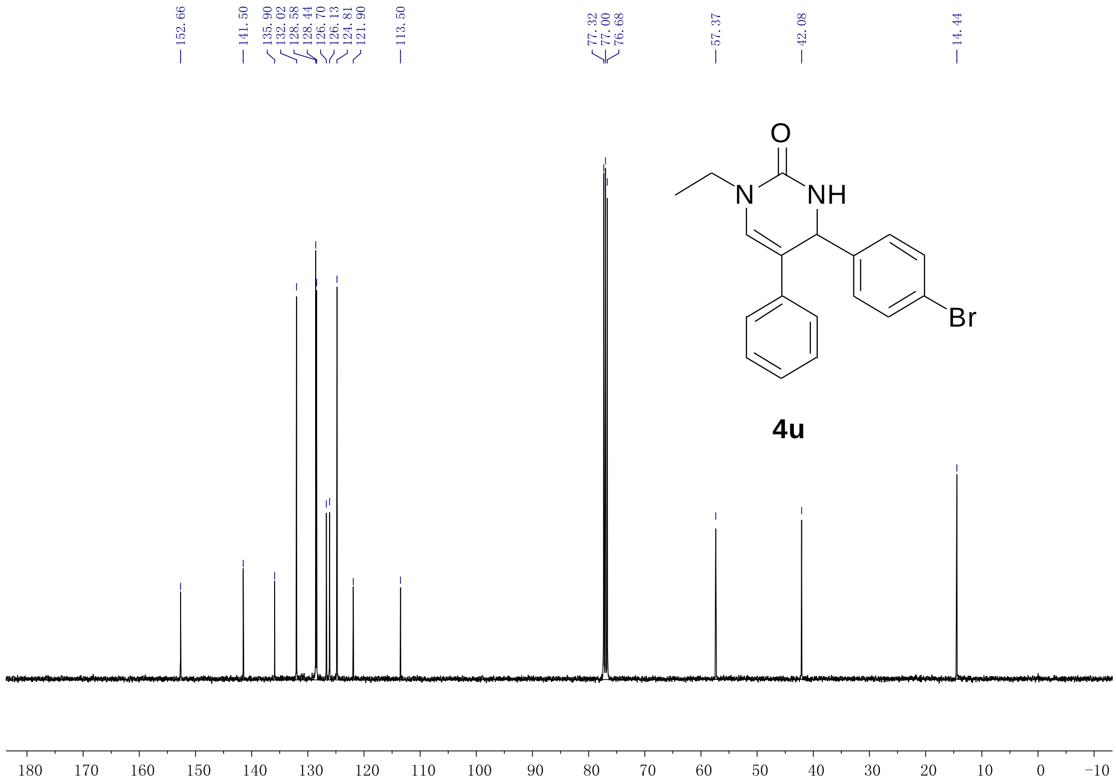
152.66
141.50 135.90 132.02 128.58 128.44 126.70 126.13 124.81 121.90
113.50
77.32 77.00 76.68
57.37
42.08
14.44
O
N
NH
Br
4u

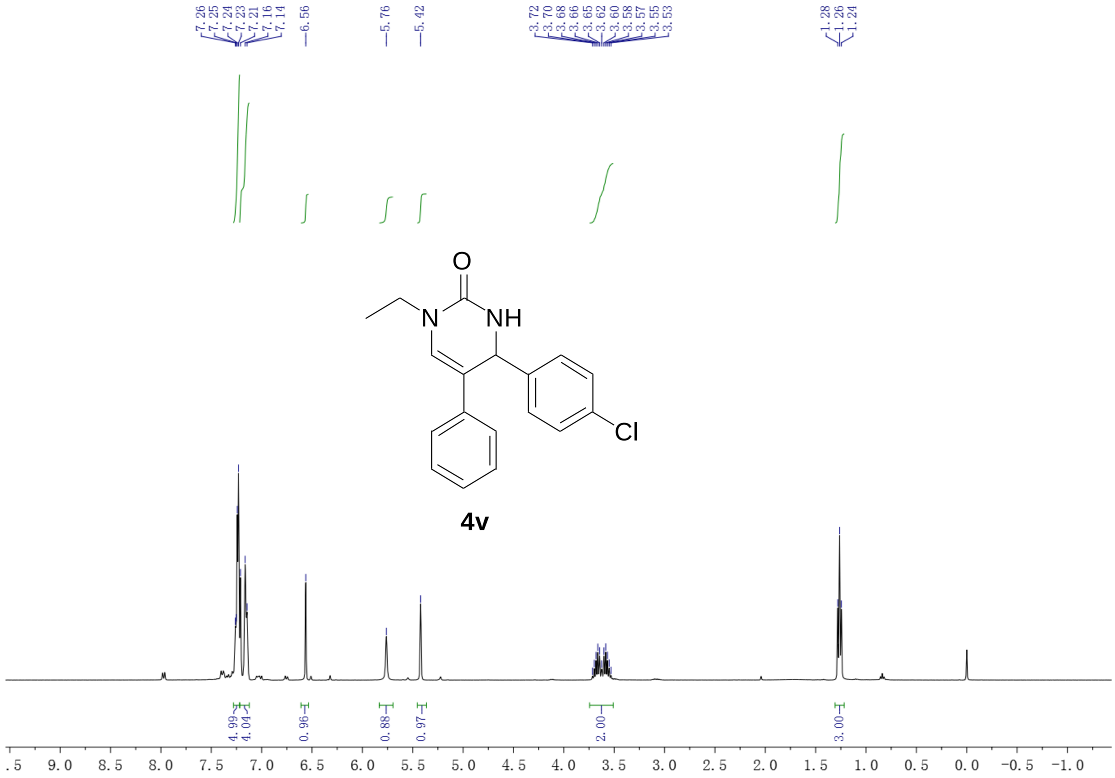
7.26
7.25
7.24
7.23
7.21
7.16
7.14
6.56
5.76
5.42
3.72
3.70
3.68
3.66
3.65
3.62
3.60
3.58
3.57
3.55
3.53
1.28
1.26
1.24
O
N
NH
Cl
4v
4.99
4.04
0.96
0.88
0.97
2.00
3.00

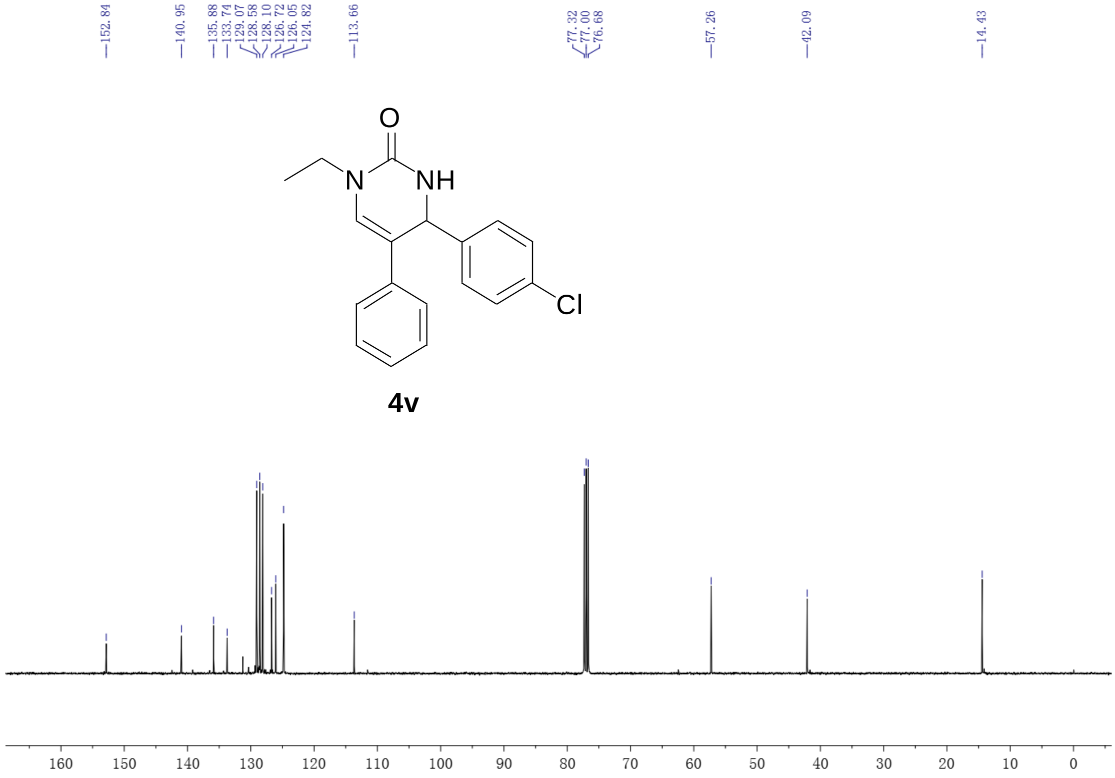
152.84
140.95
135.88
133.74
129.07
128.58
128.10
126.72
126.05
124.82
113.66
77.32
77.00
76.68
57.26
42.09
14.43
O
N
NH
Cl
4v

HPLC spectra

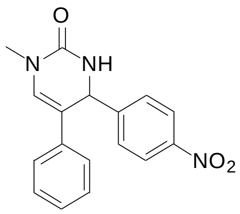

4a

77% ee. [Daicel Chiralpak AD-H, *n*-hexane / isopropanol = 80 / 20, 1.0 mL/min, λ = 254 nm]

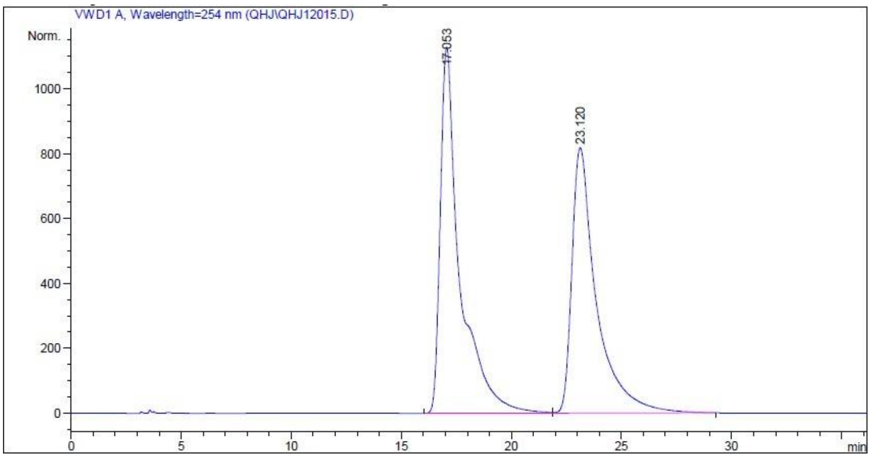

Peak #	RetTime [min]	Type	Width [min]	Area mAU *s	Height [mAU]	Area %
1	17.053	BV	0.8698	6.94847e4	1128.44250	52.3359
2	23.120	VB	1.1035	6.32821e4	818.01239	47.6641
Totals :				1.32767e5	1946.45490	

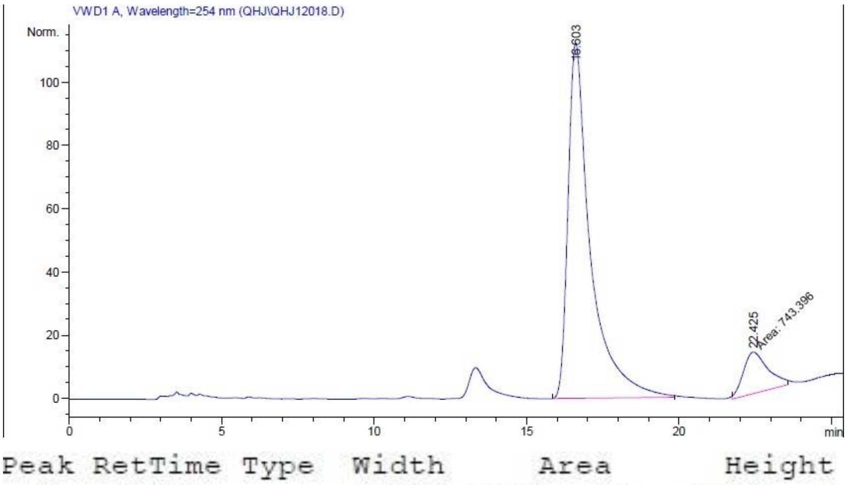

Peak #	RetTime [min]	Type	Width [min]	Area mAU *s	Height [mAU]	Area %
1	16.603	BB	0.7377	5761.65234	112.42514	88.5720
2	22.425	MM	0.9375	743.39648	13.21661	11.4280
Totals :				6505.04883	125.64175	

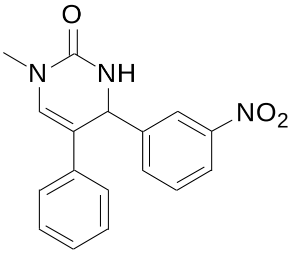

4b

75% ee. [Daicel Chiralpak AD-H, *n*-hexane / isopropanol = 80 / 20, 1.0 mL/min, λ = 254 nm]

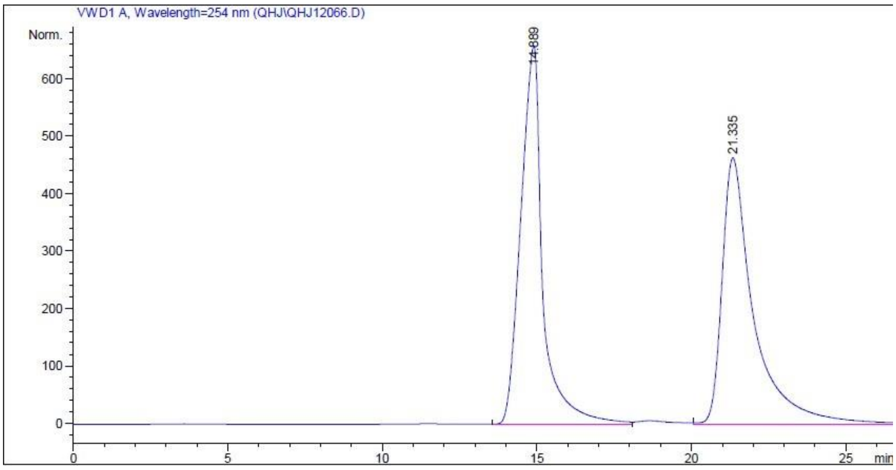

Peak #	RetTime [min]	Type	Width [min]	Area mAU *s	Height [mAU]	Area %
1	14.889	BV	0.7669	3.26258e4	658.93701	49.7644
2	21.335	VBA	1.0233	3.29348e4	463.92462	50.2356
Totals :				6.55606e4	1122.86163	

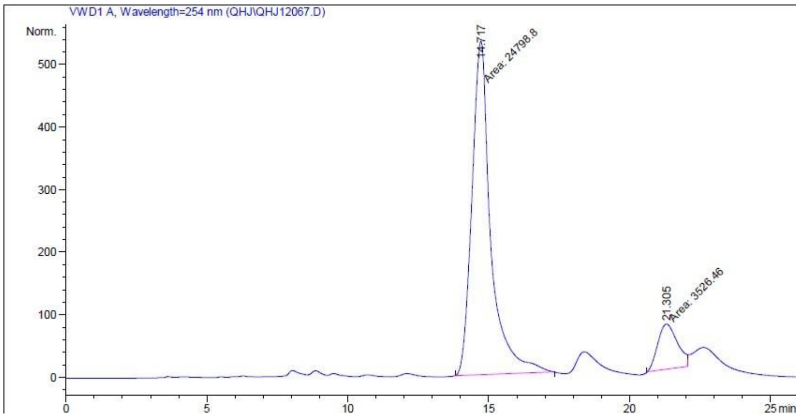

Peak #	RetTime [min]	Type	Width [min]	Area mAU *s	Height [mAU]	Area %
1	14.717	MM	0.7747	2.47988e4	533.51208	87.5501
2	21.305	MM	0.8116	3526.46167	72.41758	12.4499
Totals :				2.83252e4	605.92966	

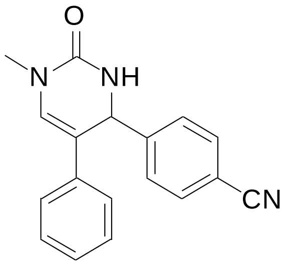

4c

64% ee. [Daicel Chiralpak AD-H, *n*-hexane / isopropanol = 80 / 20, 1.0 mL/min, λ = 254 nm]

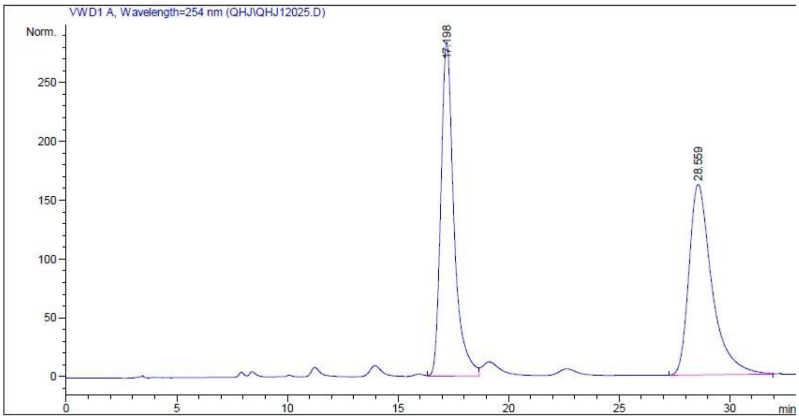

Peak #	RetTime [min]	Type	Width [min]	Area mAU *s	Height [mAU]	Area %
1	17.198	VV	0.6143	1.16938e4	284.00729	49.9626
2	28.559	BB	1.0813	1.17113e4	162.17480	50.0374
Totals :				2.34051e4	446.18210	

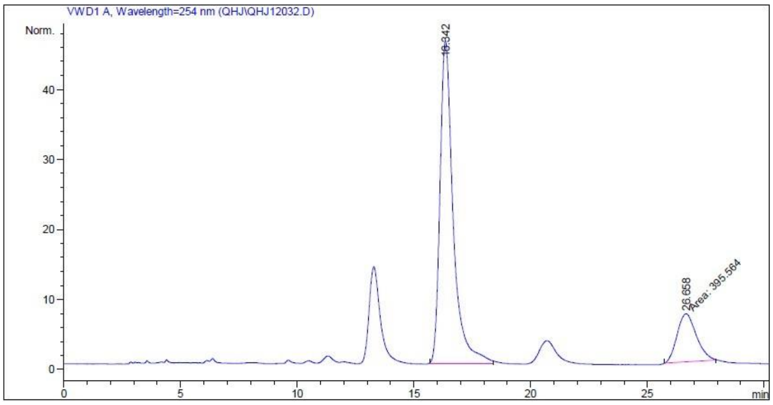

Peak #	RetTime [min]	Type	Width [min]	Area mAU *s	Height [mAU]	Area %
1	16.342	BB	0.5914	1809.20642	46.14186	82.0587
2	26.658	MM	0.9567	395.56396	6.89142	17.9413
Totals :				2204.77039	53.03328	

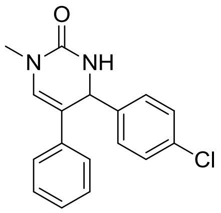

4d

32% ee by chiral HPLC analysis [Daicel Chiralpak AD-H, *n*-hexane / isopropanol = 90/10,0.8 mL/min, λ = 254 nm, *t* (major) = 29.13min, *t* (minor) = 31.84 min].

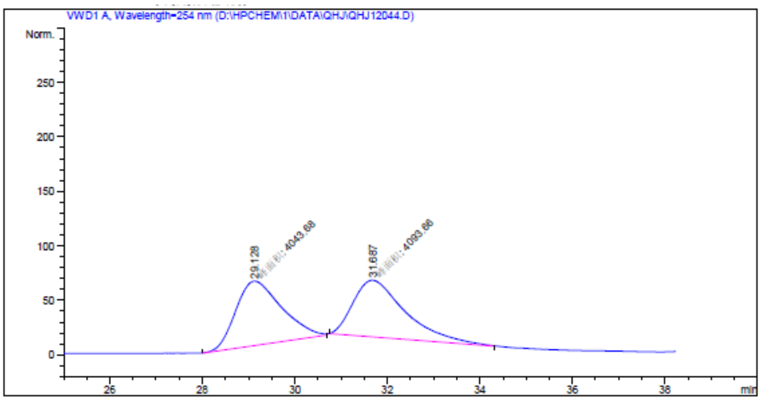

面积百分比报告

排序 : 信号
乘积因子 : 1.0000
稀释因子 : 1.0000
内标使用乘积因子和稀释因子

信号 1: VWD1 A, Wavelength=254 nm

峰 #	保留时间 [min]	类型	峰宽 [min]	峰面积 mAU *s	峰高 [mAU]	峰面积 %
1	29.128	MM	1.1421	4043.67993	59.01037	49.6929
2	31.687	MM	1.3107	4093.66016	52.05369	50.3071

总量 : 8137.34009 111.06406

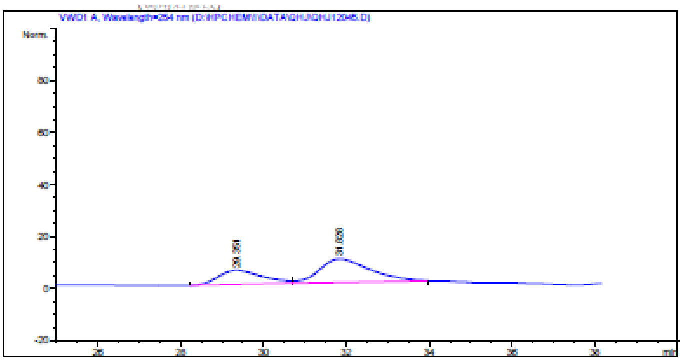

面积百分比报告

排序 : 信号
乘积因子 : 1.0000
稀释因子 : 1.0000
内标使用乘积因子和稀释因子

信号 1: VWD1 A, Wavelength=254 nm

峰 #	保留时间 [min]	类型	峰宽 [min]	峰面积 mAU *s	峰高 [mAU]	峰面积 %
1	29.351	BV	0.8643	397.15692	5.43445	33.8463
2	31.828	VB	1.0489	776.25714	9.08826	66.1537

总量 : 1173.41406 14.52271

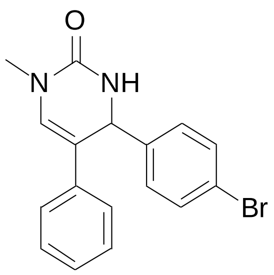

4e

53% ee. [Daicel Chiralpak AD-H, *n*-hexane / isopropanol = 80 / 20, 1.0 mL/min, λ = 254 nm]

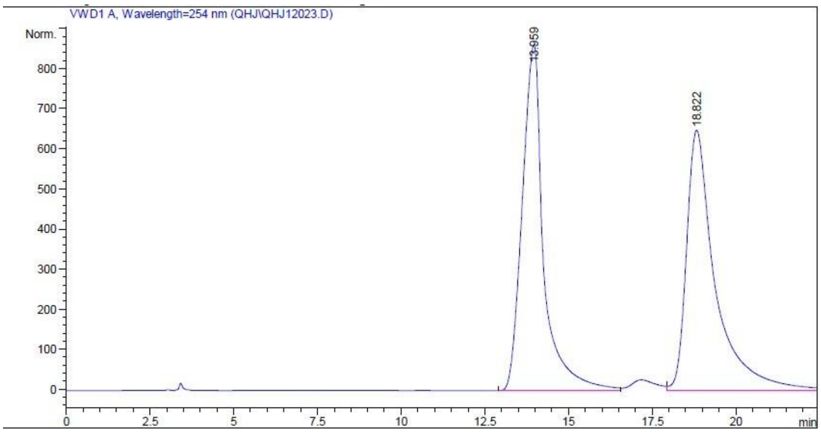

Peak #	RetTime [min]	Type	Width [min]	Area mAU *s	Height [mAU]	Area %
1	13.959	BV	0.6734	3.84118e4	863.40009	49.6912
2	18.822	VBA	0.8672	3.88892e4	649.72180	50.3088

Totals : 7.73011e4 1513.12189

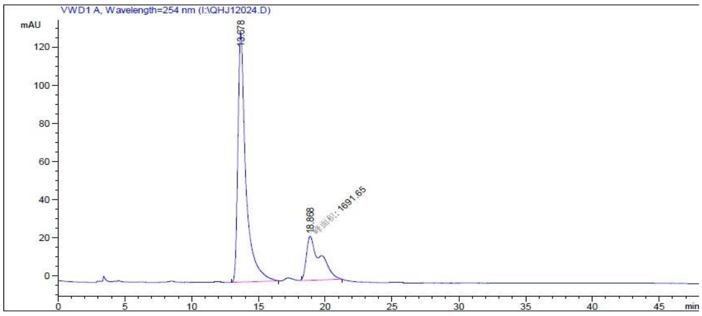

峰 #	保留时间 [min]	类型	峰宽 [min]	峰面积 mAU *s	峰高 [mAU]	峰面积 %
1	13.678	BB	0.6000	5445.21436	130.98067	76.2971
2	18.868	MM	1.2166	1691.64575	23.17445	23.7029

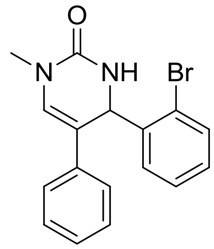

4f

20% ee by chiral HPLC analysis [Daicel Chiralpak AD-H, *n*-hexane / isopropanol = 90/10, 0.8 mL/min, λ = 254 nm, *t* (major) = 29.30 min, *t* (minor) = 31.37 min].

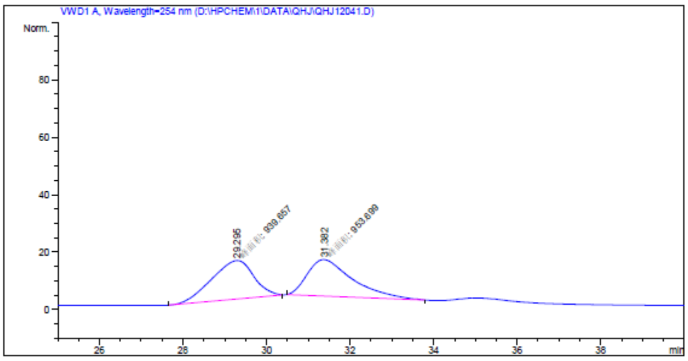

面积百分比报告

排序 : 信号
乘积因子 : 1.0000
稀释因子 : 1.0000
内标使用乘积因子和稀释因子

信号 1: VWD1 A, Wavelength=254 nm

峰 #	保留时间 [min]	类型	峰宽 [min]	峰面积 mAU *s	峰高 [mAU]	峰面积 %
1	29.295	MM	1.1731	939.65710	13.34975	49.6292
2	31.382	MM	1.2570	953.69946	12.64469	50.3708

总量 : 1893.35657 25.99444

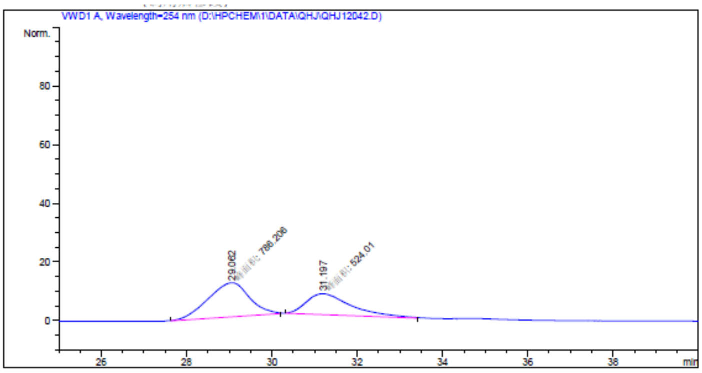

面积百分比报告

排序 : 信号
乘积因子 : 1.0000
稀释因子 : 1.0000
内标使用乘积因子和稀释因子

信号 1: VWD1 A, Wavelength=254 nm

峰 #	保留时间 [min]	类型	峰宽 [min]	峰面积 mAU *s	峰高 [mAU]	峰面积 %
1	29.062	MM	1.1321	786.20575	11.57461	60.0058
2	31.197	MM	1.2169	524.00977	7.17682	39.9942

总量 : 1310.21552 18.75143

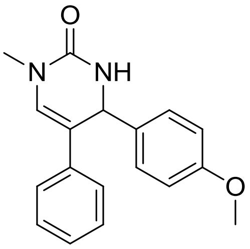

4j

33% ee by chiral HPLC analysis [Daicel Chiralpak AD-H, *n*-hexane / isopropanol = 90/10, 0.8 mL/min, λ = 254 nm, *t* (major) = 43.37 min, *t* (minor) = 48.50 min]

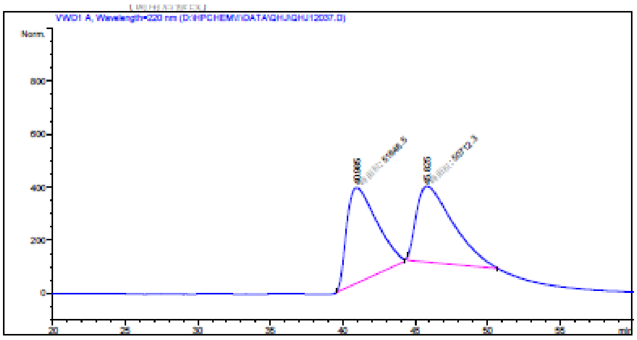

面积百分比报告

排序 : 信号
乘积因子 : 1.0000
稀释因子 : 1.0000
内标使用乘积因子和稀释因子

信号 1: VWD1 A, Wavelength=220 nm

峰 #	保留时间 [min]	类型	峰宽 [min]	峰面积 mAU *s	峰高 [mAU]	峰面积 %
1	40.985	MM	2.3898	5.16465e4	360.18765	50.4564
2	45.825	MM	2.9569	5.07123e4	285.83899	49.5436
总量 :				1.02359e5	646.02664	

(调用后修改)

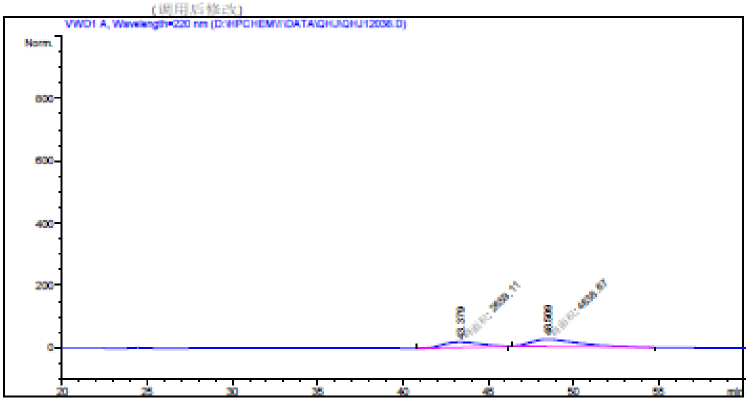

面积百分比报告

排序 : 信号
乘积因子 : 1.0000
稀释因子 : 1.0000
内标使用乘积因子和稀释因子

信号 1: VWD1 A, Wavelength=220 nm

峰 #	保留时间 [min]	类型	峰宽 [min]	峰面积 mAU *s	峰高 [mAU]	峰面积 %
1	43.379	MM	2.5333	2659.10962	17.49437	36.4372
2	48.509	MM	3.3599	4638.67139	23.01027	63.5628
总量 :				7297.78101	40.50464	

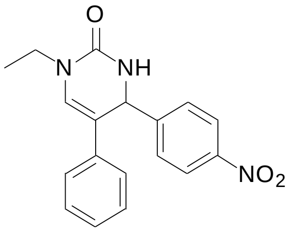

4r

32% ee. [Daicel Chiralpak AS-H, *n*-hexane / isopropanol = 80 / 20, 0.8 mL/min, λ = 254 nm]

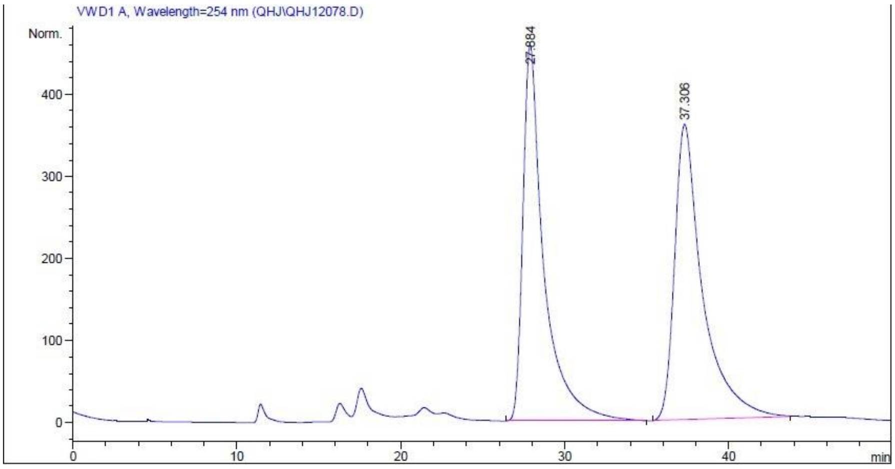

Peak #	RetTime [min]	Type	Width [min]	Area mAU *s	Height [mAU]	Area %
1	27.884	PP	1.2738	4.08288e4	457.63019	48.8943
2	37.306	PB	1.7210	4.26754e4	359.94171	51.1057

Totals : 8.35043e4 817.57190

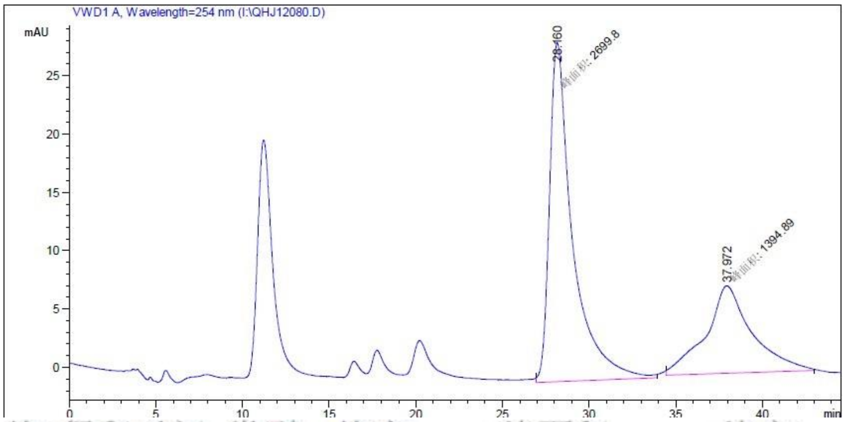

峰 #	保留时间 [min]	类型	峰宽 [min]	峰面积 mAU *s	峰高 [mAU]	峰面积 %
1	28.160	MM	1.5473	2699.80127	29.08039	65.9342
2	37.972	MM	3.1005	1394.88831	7.49815	34.0658
